# Direct measurement of cruising and burst swimming speeds of the shortfin mako shark (*Isurus oxyrinchus*) with estimates of field metabolic rate

**DOI:** 10.1111/jfb.15475

**Published:** 2023-07-04

**Authors:** Matt J. Waller, Nuno Queiroz, Ivo da Costa, Tiago Cidade, Bruno Loureiro, Freya C. Womersley, Jorge Fontes, Pedro Afonso, Bruno C. L. Macena, Alexandra Loveridge, Nicolas E. Humphries, Emily J. Southall, David W. Sims

**Affiliations:** ^1^ Marine Biological Association The Laboratory Plymouth UK; ^2^ Ocean and Earth Science, National Oceanography Centre Southampton University of Southampton Southampton UK; ^3^ CIBIO/InBIO, Universidade do Porto Campus Agrário de Vairão, Rua Padre Armando Quintas Vairão Portugal; ^4^ Institute of Marine Research – IMAR Universidade dos Açores Horta Portugal; ^5^ Institute of Marine Sciences – OKEANOS University of the Azores Horta Portugal

**Keywords:** accelerometry ecophysiology, endothermy, lamnidae, swimming performance

## Abstract

The shortfin mako shark is a large‐bodied pursuit predator thought to be capable of the highest swimming speeds of any elasmobranch and potentially one of the highest energetic demands of any marine fish. Nonetheless, few direct speed measurements have been reported for this species. Here, animal‐borne bio‐loggers attached to two mako sharks were used to provide direct measurements of swimming speeds, kinematics and thermal physiology. Mean sustained (cruising) speed was 0.90 m s^−1^ (±0.07 s.d.) with a mean tail‐beat frequency (TBF) of 0.51 Hz (±0.16 s.d.). The maximum burst speed recorded was 5.02 m s^−1^ (TBF_max_ = 3.65 Hz) from a 2 m long female. Burst swimming was sustained for 14 s (mean speed = 2.38 m s^−1^), leading to a 0.24°C increase in white muscle temperature in the 12.5 min after the burst. Routine field metabolic rate was estimated at 185.2 mg O_2_ kg^−1^ h^−1^ (at 18°C ambient temperature). Gliding behaviour (zero TBF) was more frequently observed after periods of high activity, especially after capture when internal (white muscle) temperature approached 21°C (ambient temperature: 18.3°C), indicating gliding probably functions as an energy recovery mechanism and limits further metabolic heat production. The results show shortfin mako sharks generally cruise at speeds similar to other endothermic fish – but faster than ectothermic sharks – with the maximum recorded burst speed being among the highest so far directly measured among sharks, tunas and billfishes. This newly recorded high‐oxygen‐demand performance of mako sharks suggests it may be particularly vulnerable to habitat loss due to climate‐driven ocean deoxygenation.

## 1 INTRODUCTION

Activity is energetically costly, with processes such as locomotion comprising one of the largest sources of energy expenditure in the majority of mobile species (Lawson *et al*., 2019). This necessitates that animals optimise movement strategies to maximise the energy gained from food resources *vs*. the energy expended in its collection, such as during prey capture (Papastamatiou *et al*., 2018; Sims, 2003). The selection of movement strategies, in terms of speed, power output and duration of active locomotion, therefore has important implications for foraging, growth and fitness (Weihs & Webb, 1983).

Determining the activity levels of free‐ranging animals allows the estimation of energetic costs of different behaviours, which can in turn provide a better understanding of species physiology, ecology and evolution (Lawson *et al*., 2019; Louw, 1993). Knowledge of the physiological ecology of large pelagic fishes is particularly important for revealing mechanisms that underpin habitat suitability, species distributions and ecological functioning (Bernal *et al*., 2010), information which can inform the conservation of threatened populations (McKenzie *et al*., 2016; Vedor *et al*., 2021). For example, behaviours and activity levels occurring at fine spatio‐temporal scales are likely to be key drivers of trophic interactions, which will affect habitat selection and thereby distributions (Andrzejaczek *et al*., 2019; Bowlby *et al*., 2022), ultimately driving spatial overlap with anthropogenic activities such as fishing (Queiroz *et al*., 2016; Queiroz *et al*., 2019; Queiroz *et al*., 2021). Nonetheless, due to the inherent difficulties in studying large animals in open‐ocean environments, data on the fine‐scale behaviours and energetics of apex predators such as sharks are often lacking (Lawson *et al*., 2019; McKenzie *et al*., 2016; Payne *et al*., 2015; Sims, 2003). This is principally due to the logistical challenges associated with attaching devices to oceanic sharks for enough time to record body movements and activity level at high temporal resolution, coupled with the consequent retrieval of resulting large volumes of data from remote destinations often reached by highly migratory sharks (Lawson *et al*., 2019; Sims, 2010).

The shortfin mako shark (*Isurus oxyrinchus* Rafinesque, 1809), hereafter referred to as mako, is a highly active predatory species found globally in temperate and tropical pelagic habitats (Francis *et al*., 2019; Nasby‐Lucas *et al*., 2019; Vaudo *et al*., 2017). Diet studies show that mako sharks typically predate fast‐moving teleost fish, including mackerel, sauries, tunas and billfishes (Biton‐Porsmoguer *et al*., 2017; Maia *et al*., 2006; Stillwell & Kohler, 1982; Wood *et al*., 2009). Hunting highly active species by pursuit predation requires the mako to match or overtake its prey's swimming speed, indicating that very fast swimming speeds are necessary. Mako burst speeds of up to 35 km h^−1^ (9.8 m s^−1^) have been estimated based on calculating the probable escape velocity required to attain the observed height above water reached by leaping makos when hooked by rod‐and‐line angling (Carey & Teal, 1969). These high estimated swimming speeds are consistent with results from respirometry studies, which show mako sharks to have some of the highest oxygen demands, per unit of body mass, of any marine fish (Bernal *et al*., 2012; Graham *et al*., 1990; Sepulveda *et al*., 2007). Makos possess a range of physiological adaptations associated with such elevated levels of aerobic activity, including regional endothermy (Bernal *et al*., 2012) and specialised gill morphologies (Wegner *et al*., 2010). However, few direct measurements of swimming speeds attained by free‐living mako sharks in their natural environment have been reported.

Given their elevated metabolic rates and oxygen demands, mako sharks are likely to be highly sensitive to environmental changes linked to anthropogenic climate change, such as declining dissolved oxygen (DO) concentrations in the ocean (ocean deoxygenation) (Abascal *et al*., 2011; Vedor *et al*., 2021). Mako sharks have also been of significant commercial importance for meat and fins as a result of high spatial and temporal overlap with commercial fishing effort across their range (Queiroz *et al*., 2019). High rates of fishing mortality, coupled with low fecundity, slow growth and late age to sexual maturity have resulted in an estimated decline in relative abundance of about 40% over the past half century (Pacoureau *et al*., 2021), with the species now listed as globally “endangered” by the IUCN Red List (Rigby *et al*., 2019). As such, data on the fine‐scale movements, activity levels and field energetics of this species are needed to better understand the link between physiological function and ecological performance. This is especially important with respect to how this may alter the selection of habitats by sharks and which may shift the intensity of shark–fisheries interactions under current and future climatic regimes (Vedor *et al*., 2021).

The incorporation of tri‐axial accelerometers into bio‐logging systems allows for measurements of behavioural metrics down to sub‐second scale (>100 Hz) (Cade *et al*., 2021; Fontes *et al*., 2022). These systems have been applied to multiple aspects of shark ecology, providing insights into predator–prey interactions (Andrzejaczek *et al*., 2019; Watanabe *et al*., 2019a), swimming kinematics (Nakamura *et al*., 2011; Payne *et al*., 2016; Watanabe *et al*., 2019b) and mating behaviours (Whitney *et al*., 2010). Accelerometry‐derived metrics, such as dynamic body accelerations, may also serve as proxies for energy expenditure (Metcalfe *et al*., 2016; Wilson *et al*., 2020). While the use of telemetry data to estimate the metabolic rates of free‐swimming pelagic sharks goes back to at least the 1980s (Carey *et al*., 1982), the incorporation of accelerometers and other instruments such as speed and pressure sensors into bio‐logging packages represents a valuable recent tool for inferring field metabolic rates (FMR) for large shark species in their natural environments (Lawson *et al*., 2019). Such methods have already been applied to shark ecology studies, including the energetic costs of feeding behaviours in whale sharks (*Rhincodon typus*) (Cade *et al*., 2020) and the cost of transport of white sharks (*Carcharodon carcharias*) (Watanabe *et al*., 2019b). Estimation of metabolic rate parameters such as routine metabolic rate (RMR) allows for a more thorough understanding of key ecological parameters such as energy budgets and trophodynamics (Sepulveda *et al*., 2007). Nonetheless, basic direct measurements of swimming speeds in combination with other physiological measurements such as internal temperature are lacking for many shark species precluding deeper insights into their physiological ecology under natural conditions.

This study uses animal‐borne accelerometers and speed sensors to investigate the *in situ* movements, behaviour and energetics of two free‐swimming mako sharks. The aims were to (a) directly measure sustained swimming and burst speeds of makos in their natural environment; (b) quantify behavioural metrics such as tail‐beat frequency (TBF) and angles of the longitudinal body axis relative to horizontal swimming and determine how these parameters change through time and different swimming modes; (c) directly measure internal muscle temperatures and how these are influenced by swimming behaviours; and (d) provide the first estimates for FMR from free‐swimming mako sharks.

## 2 MATERIALS AND METHODS

### 2.1 Bio‐logging tags

**FIGURE 1:**
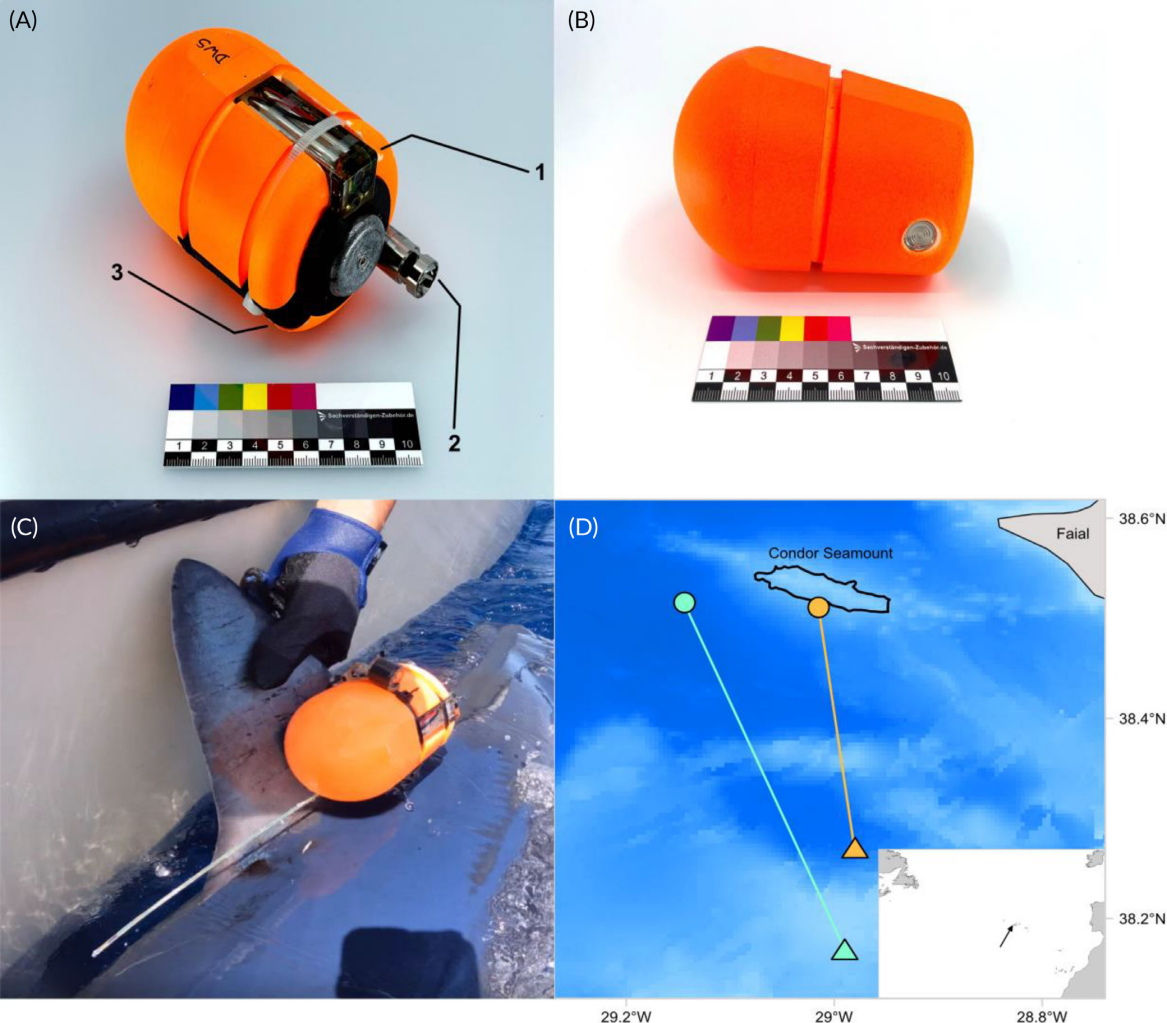
Bio‐logging tags, tagging and pop‐up locations of shortfin mako sharks (*Isurus oxyrinchus*). (a) MS (multi‐sensor) tag; arrow 1 shows the DCL400M camera, arrow 2 shows the ORI1300 3MPD3GT data logger and arrow 3 shows the internal temperature logger. (b) DOME (Dissolved Oxygen Measuring) tag. (c) Attachment of MS tag to S1 (note this photo was taken before insertion of the internal temperature probe). (d) Tagging and pop‐up locations of both tags. Circles denote tagging locations taken from the tagging vessel's GPS (global positioning system) during tagging, and triangles denote pop‐up locations from first GPS positions transmitted by tags. Black line shows the position of the Condor Seamount. Grey denotes land (island of Faial, Azores). Inset in panel (d) shows the North Atlantic, with Faial shown by a black arrow. Maps in panel (d) were produced in Arc GIS Pro 2.7.7 (ESRI, Redland, CA, USA); bathymetric data were taken from www.gebco.net; darker colours correspond to deeper water. 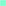 Shark S1; 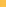 Shark S2.

Two bio‐logging tag types were developed for use in this study. The first, a multi‐sensor (MS) tag, consisted of an ORI1300 3MPD3GT data logger (Little Leonardo Corp., Tokyo, Japan) capable of recording tri‐axial accelerometry and magnetometry (for fine‐scale, 3D movements at 1–30 Hz) in addition to swimming speed (0.2–10 m s^−1^, resolution: 0.05 m s^−1^), pressure (range: 0–1300 m, resolution: 0.4 m) and water temperature (−10 to 50°C, resolution: 0.1°C); a DCL400M video camera (Little Leonardo) for recording the environment visible ahead of the shark and possible prey encounters; and a LAT2810S internal temperature sensor (Lotek, Newmarket, Canada) for recording dorsal internal (white muscle) temperature. The tag also contained a GPS (global positioning system) unit for geolocation after programmed release from a shark. A resin‐based flotation package was designed to house the sensors (120 mm in length and 80 mm in diameter, weight in air = 491 g and weight in water = 60 g) (Figure 1a). The MS tag was held in position using a timed‐release cable tie against a 3D‐printed polylactic acid (PLA) mounting plate that was attached to the base of a shark's fin (see the “Shark tagging” section). The electronic timed release, operated by a timer attached to a small charge within the cable tie, enabled the tag to detach from a shark at the pre‐programmed time, float to the surface and transmit its position *via* GPS satellites to allow recovery by a vessel. The second tag used was a Dissolved Oxygen Measuring (DOME) archival tag, which consisted of a tri‐axial accelerometer recording at 1–30 Hz and environmental sensors that record pressure (depth), water temperature and oxygen data at intervals from 1 to 60 s (Figure 1b). Oxygen data were not considered further in this study. Pressure (range: 0–6000 m, resolution: 0.20 m) and temperature (range: 0–50°C, resolution: 0.10°C) were recorded by an ultra‐compact, high‐pressure OEM sensor (Series 6LHP, Keller, Winterthur, Switzerland). As with the MS tag, the DOME archival tag was secured to a PLA plate *via* a timed‐release mechanism and consisted of a GPS unit for geolocation and recovery by a vessel. The electronic components were housed within a resin‐based flotation package that was 135 mm in length, 85 mm in diameter and 505 g (total weight) in air (weight in water = 30 g). For both tags, accelerometer, magnetometer and gyroscopic sensors recorded data at 20 Hz, with all other sensors recording at 1 Hz.

### 2.2 Speed sensor calibration

During deployment the data logger in the MS tag was set to record speed using the factory calibration coefficient which automatically converts the revolutions of the propeller to speed (m s^−1^). To establish the relationship between this derived speed and true speed through the water, a manual calibration of the speed sensor was conducted by mounting it on a torpedo‐shaped body (to mimic attachment to a shark), which was then towed at the side of a small boat at *c*. 2 m depth (ahead of and below the boat's wake), in “runs” along a measured test distance of 400 m. Calibration runs were conducted within the Leixões seaport, Porto, Portugal, in very low‐wind‐speed conditions close to slack water to reduce both surface and at‐depth water movements that could affect recorded speeds (Supporting Information Figure S1; see Data S1 – Tag calibration). Two fixed points were chosen within the confines of the harbour walls of the seaport between which calibration runs were conducted. Runs were conducted at nominal speeds of 1.0, 2.0, 3.0 and 4.0 m s^−1^, with two runs conducted at each speed in opposite directions to reduce any potential effects of residual surface or at‐depth water movements. Through‐the‐water speeds recorded by the MS tag were compared to vessel speeds that were measured with an RTK Facet L‐Band GPS (SparkFun, Boulder, CO, USA), using mode “GNSS Positioning” which is accurate to within *c*. 0.30 m. As the vessel speed varied during the run distance, for example, as the vessel was brought up to the test speed, the vessel speed was determined from the least variable section of each run's speed time series. The point at which the speed of the vessel became stable was determined using an “at most one change” change‐point analysis, which is a binary method capable of identifying up to one change point within a data set (Killick & Eckley, 2014). This was used to define the sections of each run that were most reliable for use in calibration analysis, that is, the period from when the vessel first reached the target speed to when it decelerated below the target. A linear regression forced through the origin of vessel speed against logger‐recorded speeds of the MS tag was then calculated using the selected constant‐speed data (Supporting Information Figure S2). The slope of this linear regression was used to correct the speed recorded by the logger attached to a shark. The swimming speed of the shark fitted with the DOME archival tag was estimated using the slope and intercept of a linear regression of the relationship between calibrated speed and TBF derived from the shark fitted with the MS tag.

### 2.3 Shark tagging

Mako sharks were caught on a 4 km drifting longline set by *RV Arquipelago* (Universidade dos Açores) above the slope of the Condor seamount *c*. 10 nautical miles (nmi) southwest of Faial Island, Azores, Portugal, on 14 June 2022. The longline comprised 140 hooks baited with mackerel (*Scomber scombrus*) that were set between 20 and 50 m depth with a soak time of 3.5 h. Two mako sharks from those caught were tagged: a female (S1) of 197 cm total length (TL) was fitted with the MS tag and a male (S2) of 199 cm TL with the DOME archival tag. Sharks captured on the longline were kept in the water and moved alongside a 7 m long fibreglass tagging vessel with low gunnels. Sharks remained in the water at all times and were secured near the tagging vessel's bow by the wire leader attached to the circle hook in the jaw and aft by rope loop passed around the caudal peduncle to reduce shark movements during tagging. Once the shark was secured, the vessel was moved slowly forward for *c*. 1 min to pass a flow of clean sea water over the shark's gills, before stopping for tagging to occur. Before tagging, the shark's fork and TL were measured, and sex recorded. An MS or DOME archival tag was secured to the base of the first dorsal fin by two 6 mm pins built into the PLA mounting plate that were passed through the fin and secured on the opposite side of the fin to the tag with soft, rubber washers and nylon‐locking nuts (Figure 1c). In the case of the MS tag, the internal temperature sensor was placed into the dorsal white muscle to a depth of *c*. 5–6 cm by making a small incision in the skin and gently inserting the sensor (2 mm diameter) and connecting cable (1–2 mm diameter) into the muscle. The sensor was placed into the dorsum 3 cm lateral to the posterior emargination of the first dorsal fin. Tagging procedures were completed within 5–7 min, whereupon the vessel moved slowly forward again for 1–3 min to aerate the gills during which time the caudal fin loop was removed. Steady swimming movements of the caudal fin returned immediately, and when the hook was removed, the sharks were released. The timed release of the MS tag was set to 09.00 hours local time the following day, with the DOME tag set to release at 07.00 hours local time, 2 days post tagging. At the predetermined time the cable fixing the tag to the PLA mounting plate was released, and the tag floated to the surface. The MS tag commenced GPS transmissions immediately upon release. The DOME archival tag used a timer to begin satellite transmissions: after 45 min at the surface, the tag switched to satellite mode and made repeated transmissions to satellites providing coordinates of its location. Both tags were then located and recovered by a vessel.

### 2.4 Animal ethical statement

The procedures for handling and tagging of mako sharks were performed according to national Portuguese laws for the use of vertebrates in research and were approved and authorised by the Azorean Directorate of Sea Affairs of the Autonomous Region of the Azores (AMP/2021/017), which oversees and issues permits for scientific activities, and by the Azorean Directorate of Fisheries (SAI/DRP/2021/3534), which oversees and issues permits for scientific fishing. Operations were undertaken or supervised by trained personnel at the Universidade dos Açores.

### 2.5 Data processing

Accelerometry data were analysed using a combination of Igor Pro (version 9.0, Wavemetrics Inc., Lake Oswego, OR, USA) with the *ethographer* extension (Sakamoto *et al*., 2009) and R (R Development Core Team, 2022). Before analysis, the static components of the acceleration data were separated from the dynamic components using a low‐pass filter. Static elements of the signal were used to calculate the angles of pitch (angle of the body through the longitudinal axis relative to the horizon) and roll (angle of the body through the lateral axis relative to the horizon), and these were corrected for the attachment angle of the tag to the horizontal plane of the shark's longitudinal axis (S1, MS tag: +1.98°; S2, DOME tag: +6.29°) using the method set out by Kawatsu *et al*. (2009). Three‐dimensional pseudo‐tracks of shark trajectories were constructed from acceleration, magnetometer, speed and depth data in Igor Pro using the 3D path extension (Shiomi *et al*., 2010).

### 2.6 Extraction of swimming behaviours

TBF was determined with a continuous wavelet transformation (CWT) of the dynamic acceleration of the lateral axis (Sakamoto *et al*., 2009), using a minimum cycle period of 0.1 s, a maximum cycle period of 10 s and a *W*_o_ of 10 (see Sakamoto *et al*., 2009 for details). A single tail‐beat was defined as the time taken for the caudal fin to move from one extreme lateral position to the other and back again to its starting position (Watanabe *et al*., 2019b), and TBF was expressed as the number of cycles per second (Hz). Periods of gliding (non‐powered movement) were identified using a *k*‐means clustering (*k* = 20) on the results of the CWT, with the cluster with the lowest peak identified as gliding behaviour (Nakamura *et al*., 2011).

Three swimming phases were defined for both sharks using a 60 s rolling sum centred on each point in the time series. Points with a cumulative depth change of ≥1 m were assigned as ascent or descent based on the direction of change, whereas points with a depth change of <1 m were assigned as horizontal swimming. Periods of sustained swimming (cruising) were defined as the mean swimming speed ± 2 s.d.

### 2.7 Calculation of FMR

FMR was estimated using a method adapted from Watanabe *et al*. (2019b) and based on respirometry data from juvenile mako sharks reported in Sepulveda *et al*. (2007) using the following equation:

$$\mathrm{Log}10\left(\mathrm{FMR}\right)=\log10\left(\mathrm{SMR}\right)\;+\;0.97U$$

where SMR is the whole‐body standard metabolic rate, 0.97 is the slope of the relationship between swimming speed and metabolic rate as determined by Sepulveda *et al*. (2007) and *U* is the speed in body lengths per second.

Sepulveda *et al*. (2007) calculated the SMR of a 6.1 kg mako shark in water at 18°C to be 124 mg O_2_ kg^−1^ h^−1^. This value was mass adjusted to the size of the makos tagged in this study using a scaling exponent of 0.79 (Payne *et al*., 2015). The mass of the tagged sharks was estimated using published length–weight relationship calculations from fishery‐caught individuals in the Atlantic Ocean (Supporting Information Tables S1–S3; see Data S1: Mass estimation). This mass‐adjusted SMR was further adjusted for the effects of ambient water temperature for each second in the time series using a *Q*_10_ value of 1.34 (Lowe, 2001). The effects of different scaling exponents and *Q*_10_ values on FMR estimates were explored in further detail (Supporting Information Figure S3; see Data S1: Sensitivity analysis). Instantaneous FMR was then calculated with periods of powerless gliding set to the SMR, and metabolic rate estimates were then smoothed across a minute using a rolling mean. The RMR was calculated as the mean FMR of cruising speeds.

## 3 RESULTS

Both tags successfully released from sharks S1 and S2 after deployment durations of 15.93 and 36.33 h, respectively. Tags surfaced away from Condor seamount where sharks were tagged and released but remained within the vicinity of Faial. The MS tag was first located at the surface 40.91 km southeast of where S1 was tagged and released, the DOME archival tag attached to S2 was located 29.93 km south (Figure 1d). The camera in the MS tag failed to start, and no video was recorded. Analysis of the post‐processed accelerometry data revealed the effects of environmental acceleration (*e.g*., wave action) within the top 5 m of the water column; consequently, only accelerometry data collected from below 5 m were used in further analysis.

### 3.1 Vertical space use

**FIGURE 2:**
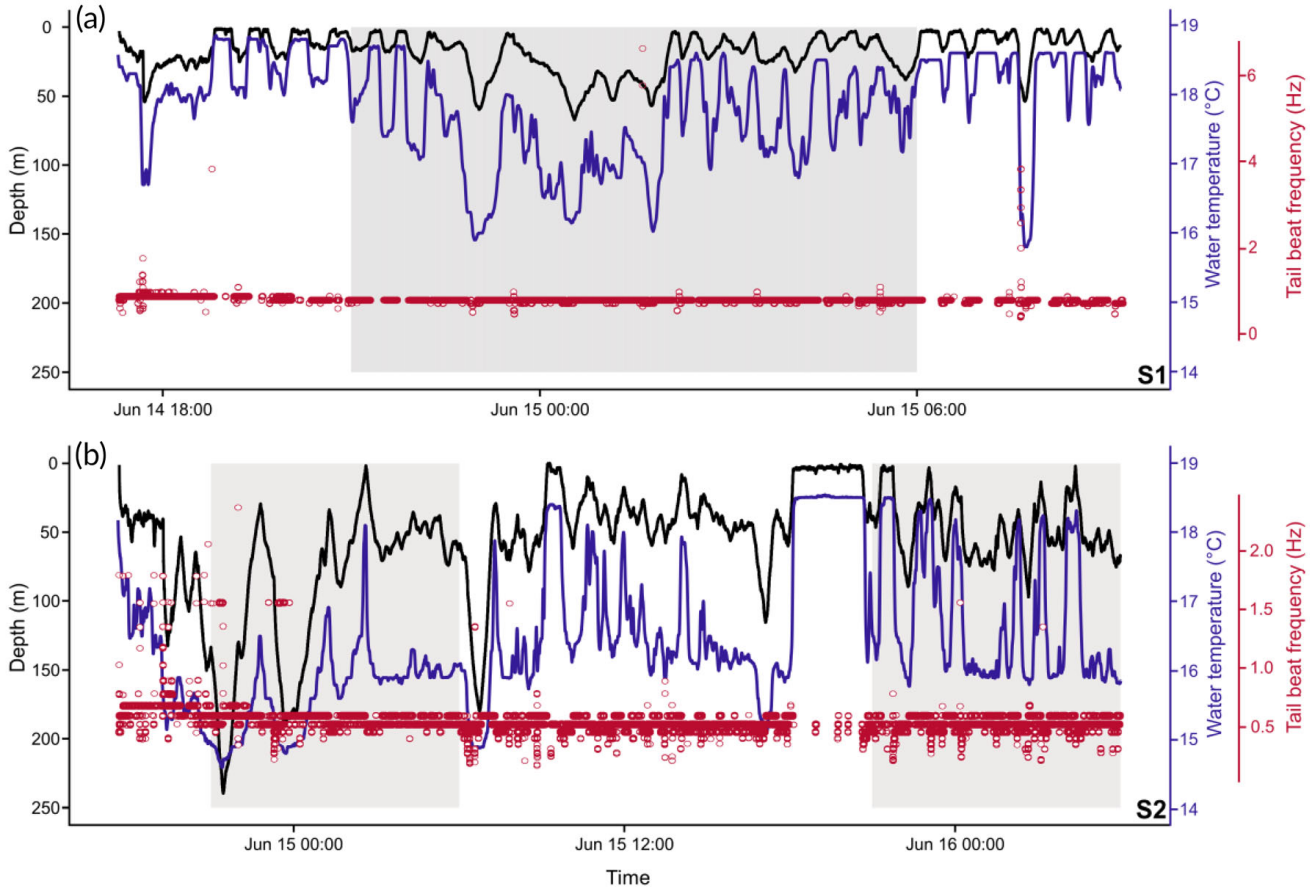
Depth and temperature profiles and instantaneous tail‐beat frequencies during powered swimming of shortfin mako sharks (*Isurus oxyrinchus*) S1 and S2 during tag deployments. Depth data were recorded at 1 Hz. Shaded areas represent periods of darkness, defined as 21.00–07.00 hours local time. Black lines represent depth use of tagged sharks (m), blue line represents ambient water temperature (°C) and red circles denote instantaneous tail‐beat frequency (Hz). (a) Shark S1, (b) shark S2

Immediately post tagging, both sharks dived to *c*. 50 m for *c*. 90 min. Vertical movements of S1 were largely confined to the upper 50 m of the water column with a mean depth of 18.5 m (±14.2 s.d.) and a maximum depth of 67.2 m (Figure 1a). Vertical movements were oscillatory in form, with time spent at the surface interspersed with generally V‐shaped dives to between 20 and 50 m before returning to the surface. S2 exhibited more extensive vertical movements with a mean depth of 57.7 m (±43.1 s.d.) and a maximum depth of 239.8 m (Figure 1b). In contrast to S1, the diving pattern of S2 showed more frequent V‐shaped dives to deeper depths for longer durations before returning to the surface or, more often, intermediate depths between 30 and 100 m. Both sharks had deeper depth distributions during the night than during the day (S1 mean depths: daytime 13.1 m ± 11.1 s.d., night‐time 22.6 m ± 15.0 s.d.; S2 mean depths: daytime 47.1 m ± 35.8 s.d., night‐time 68.4 m ± 47.1 s.d.) (Figure 2).

### 3.2 Swimming speeds and TBFs

**FIGURE 3:**
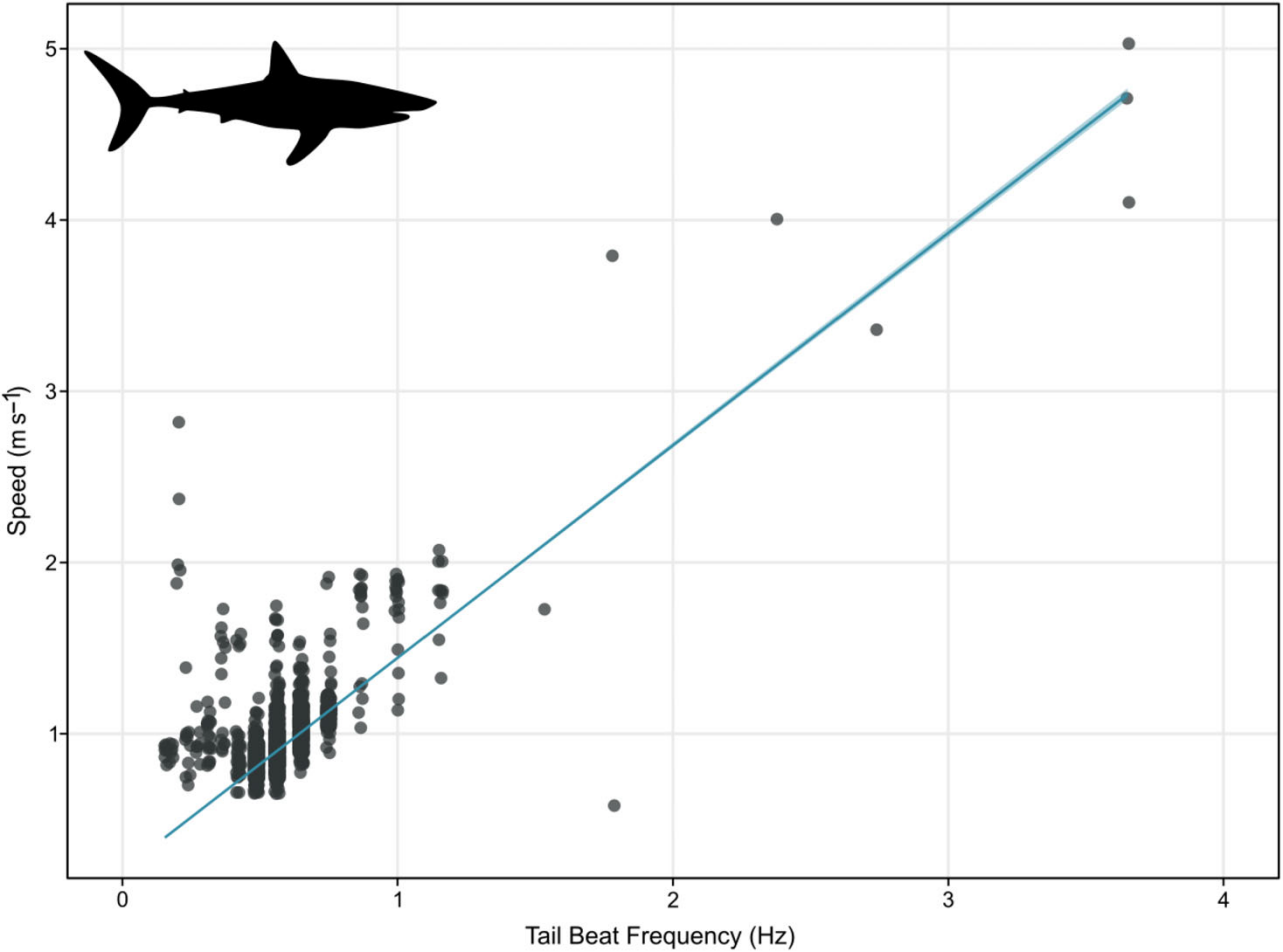
Tail‐beat frequency (TBF) *vs*. measured speed of shortfin mako shark (*Isurus oxyrinchus*) S1. Dots represent instantaneous measurements of TBF and speed for each second of deployment. Blue line represents the linear relationship between TBF and speed as calculated using a linear model (lm); shaded area represents the s.e. of this model. The fitted lm: speed = 1.24 × TBF + 0.20 with *r* ^2^ = 0.55, *P* < 0.001

The relationship between the calibrated measured speed and TBF of S1 was linear in form (*r* ^2^ = 0.55, *P* < 0.001) but showed variability even at lower speeds (Figure 3). Because the speed of S2 was not directly measured by the DOME archival tag, speed was estimated using the intercept (0.20) and slope (1.24) from the linear model between speed and TBF derived from S1 (see Figure 3 legend).

**FIGURE 4:**
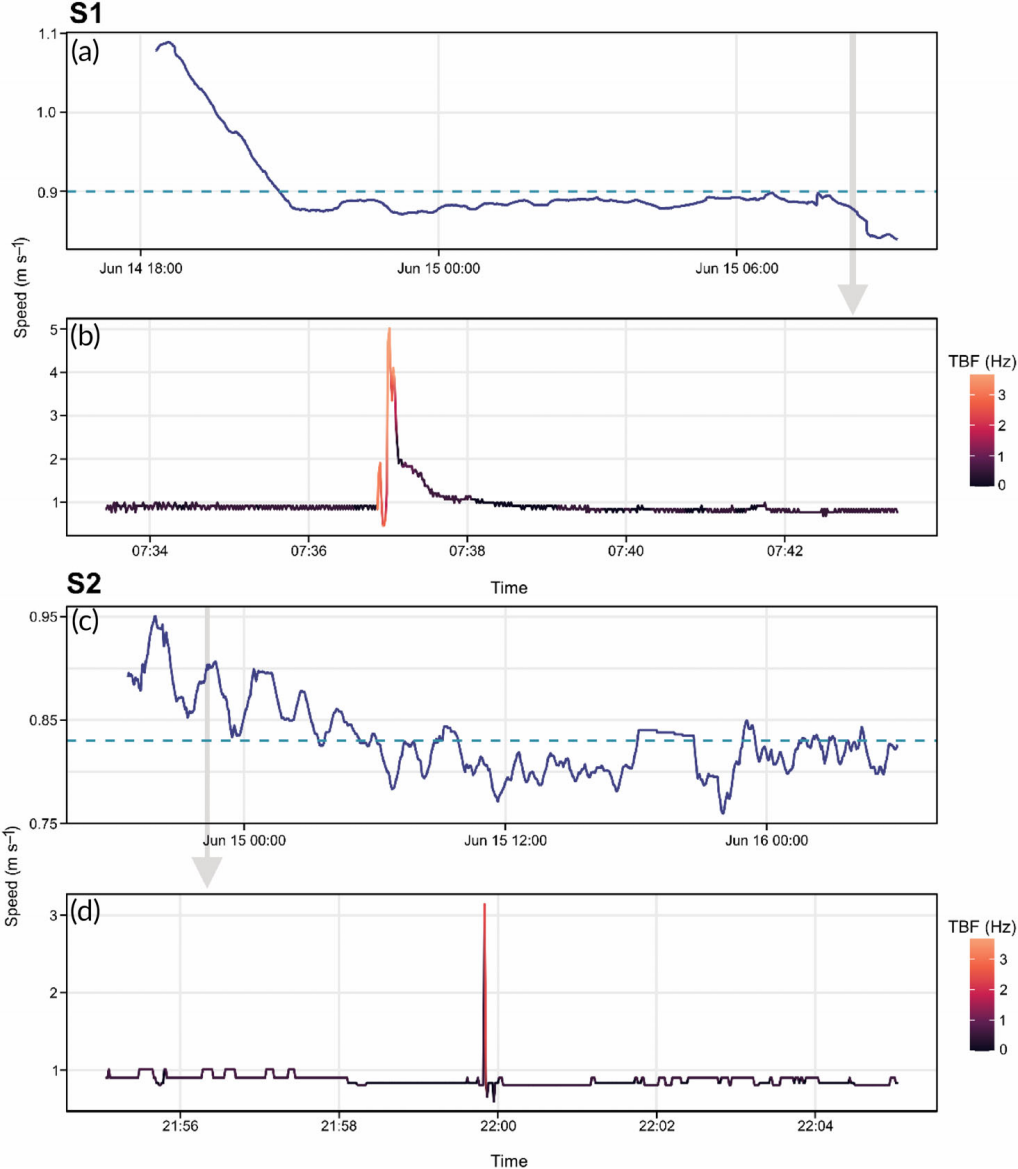
Swimming speeds of two shortfin mako sharks (*Isurus oxyrinchus*). (a and c) Swimming speeds (m s^−1^) smoothed across 60 min of S1 and S2, respectively; dashed line is the mean swimming speed of each shark. (b and d) High‐resolution plots of the maximum speeds of respective sharks. Speed of S1 was directly measured by a tag‐mounted speed sensor, and speed of S2 was estimated from the relationship between tail‐beat frequency and swimming speed. Shaded grey arrows show the period of each full track represented by high‐resolution plots.

**FIGURE 5:**
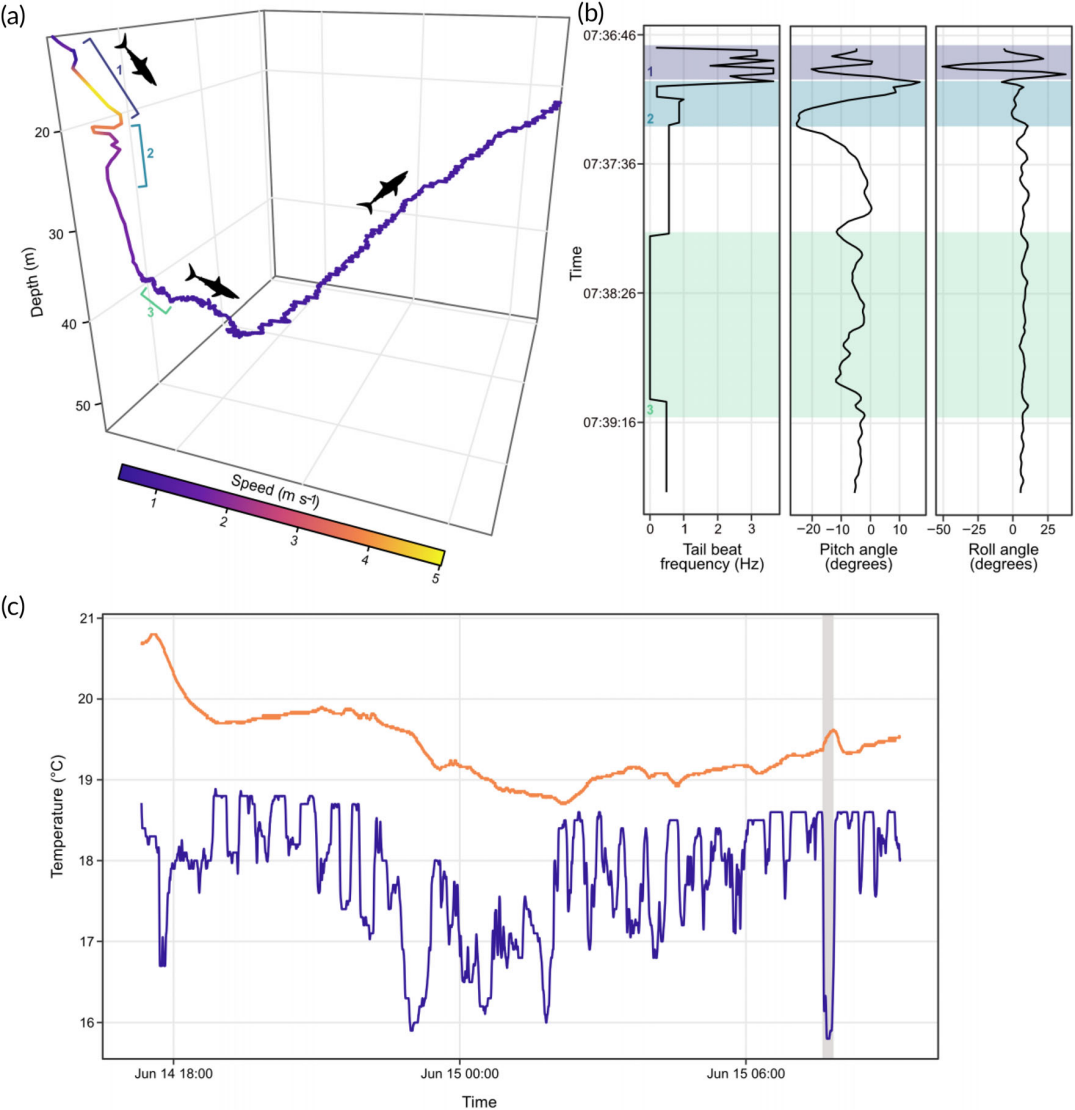
Three‐dimensional pseudo‐track of burst swimming and associated accelerometer and temperature data from shortfin mako shark (*Isurus oxyrinchus*) S1. (a) 3D pseudo‐track of burst swimming beginning at the onset of increased tail beat and ending at the end of internal muscle heating (12.5 min), coloured according to swimming speed (m s^−1^). Bracket 1 corresponds to acceleration phase; bracket 2 corresponds to period of rapid directional changes and steep descent; bracket 3 corresponds to post burst gliding period. (b) Tail‐beat frequency, pitch and roll angle of shark during burst swimming; shaded areas correspond to numbered brackets in (a). (c) Internal muscle temperature of S1 and ambient water temperature (°C) throughout entire deployment, where shaded area represents data pertaining to the 3D pseudo‐track in (a). 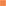, Internal temperature; 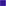, Ambient temperature

Measured speeds of S1 were elevated above mean swimming speeds for *c*. 3 h post tagging (Figure 4a). The estimated speeds of S2 were also elevated post tagging and above the mean for *c*. 9 h post tagging, although the TBF and estimated speeds during this period were variable (Figure 4c,d). Tables 1 and 2 present the speed and accelerometry metrics for each shark across dive phases. Mean speeds of S1 were consistent across different horizontal and vertical movement phases (Table 1). The cruising speed of S1 was between 0.73 and 1.08 m s^−1^ (mean: 0.90 m s^−1^ ± 0.07 s.d.) and that for S2 was estimated to be between 0.65 and 1.01 m s^−1^ (mean: 0.85 m s^−1^ ± 0.06 s.d.) (Table 1). S1 attained a maximum (burst) speed of 5.02 m s^−1^ within a burst‐swimming event that occurred within the last 2 h of the 15 h deployment (Figure 4b). This burst swimming event was also characterised by sustained increases in TBF and rapid changes in pitch and roll angles (Figure 5b). During the burst event, TBF remained elevated for 14 s, with the maximum speed reached being within 9 s of the onset of increased tail activity (mean burst speed over 14 s: 2.38 m s^−1^ ± 1.70 s.d.). This acceleration phase was followed by positive increases in pitch angle and rapid changes in horizontal direction before a steep descent, culminating in a sustained gliding period of 64 s. The maximum recorded TBF of S1 and S2 was 6.49 and 2.37 Hz, respectively, with both sharks exhibiting short, unsustained increases in TBF (1–2 s) throughout deployments. The maximum speed attained by S2 was estimated from one such brief period of increased TBF with an estimated speed of 3.85 m s^−1^. TBF decreased during descents in both individuals (Table 1) due to the higher frequency of passive gliding during descents. Nonetheless, there was little observed impact of decreased tail beat on measured swimming speeds in S1 (Table 1). When only powered swimming was considered, the mean TBF of 0.57 Hz (±0.08 s.d.) showed little variation across dive phases in S1. The TBF of powered swimming was more variable in S2 with elevated TBFs during ascents (mean: 0.55 Hz ± 0.05 s.d.) compared to descents (mean: 0.50 Hz ± 0.05 s.d.) and horizontal swimming (mean: 0.47 Hz ± 0.08 s.d.).

**TABLE 1:** Swimming speeds and tail‐beat frequencies (TBF) of two shortfin mako sharks (*Isurus oxyrinchus*) across different swimming modes and dive phases

	Shark ID	Maximum	Overall (mean ± 1 s.d.)	Cruising (mean ± 1 s.d.)	Ascent (mean ± 1 s.d.)	Descent (mean ± 1 s.d.)	Horizontal (mean ± 1 s.d.)
**Speed (m s** ^**−**1^)	S1 (F)	5.02	0.91 ± 0.09	0.90 ± 0.07	0.91 ± 0.08	0.91 ± 0.12	0.90 ± 0.07
	S2 (M)^a^	3.15	0.83 ± 0.09	0.85 ± 0.06	0.88 ± 0.06	0.78 ± 0.10	0.82 ± 0.06
TBF (Hz)	S1 (F)	6.49	0.52 ± 0.17	0.52 ± 0.16	0.58 ± 0.05	0.42 ± 0.27	0.56 ± 0.09
	S2 (M)	2.37	0.47 ± 0.15	0.52 ± 0.05	0.53 ± 0.12	0.42 ± 0.16	0.47 ± 0.13

*Note*: Cruising speeds are defined as the mean swimming speed ± 2 s.d. Dive phases are defined by 60 s rolling sum of depth changes, ascents correspond to depth changes of at least 1 m up, descents correspond to depth changes of at least 1 m down and horizontal phases correspond to portions of track where depth change is less than 1 m.

^a^ Speeds of S2 were estimated from TBF.

**TABLE 2:** Pitch and roll angles of two shortfin mako sharks (*Isurus oxyrinchus*) across different dive phases

	Shark ID	Maximum	Minimum	Overall (mean ± 1 s.d.)	Ascent (mean ± 1 s.d.)	Descent (mean ± 1 s.d.)	Horizontal (mean ± 1 s.d.)
Pitch angle (°)	S1 (F)	17.0	−35.0	0.1 ± 3.3	2.8 ± 2.1	−3.0 ± 3.2	0.2 ± 1.7
	S2 (M)	14.5	−39.2	−0.1 ± 4.4	3.9 ± 2.5	−4.2 ± 3.2	0.1 ± 2.5
Roll angle (°)	S1 (F)	37.8	−50.7	−0.1 ± 2.6	0.2 ± 2.3	−0.3 ± 3.4	−0.2 ± 2.2
	S2 (M)	14.7	−17.5	−0.1 ± 2.1	−0.2 ± 2.0	0.0 ± 2.0	−0.2 ± 2.4

*Note*: Maximum roll angle refers to the highest positive angle, and minimum roll angle refers to the maximum negative angle. Dive phases are defined by 60 s rolling sum of depth changes, ascents correspond to depth changes of at least 1 m up, descents correspond to depth changes of at least 1 m down and horizontal phases correspond to portions of track where depth change is less than 1 m.

The absolute pitch angles of ascent and descent were similar for both sharks (Table 2). The absolute maximum pitch angles occurred on downward trajectories (*i.e*., negative values). In both sharks, maximum pitch angles were observed during initial descent phases in the first hour post release. The absolute maximum pitch angles observed outside of this initial descent were −25.3° and −38.3° in S1 and S2, respectively. In S1 this corresponded to the burst swimming event (Figure 5a,b), with the shark making a steep descent at high speed. A similar relationship between pitch angle and high activity was not observed in S2, with behavioural metrics such as TBF and roll angles remaining unchanged during different levels of activity.

Maximum positive pitch angles were around half that of negative angles. The maximum positive angle observed across both sharks occurred in S1 and was recorded during the descent phase of the burst swimming event during a period of rapid horizontal movements and a reduction in descent rate (Figure 5a,b). Mean roll angles were largely consistent between dive phases in both sharks, with both sharks maintaining a level body position (<0.5°) (Table 2). Maximum roll angles of S1 were more than twice that observed in S2 and occurred during the period of sustained TBF increase associated with burst swimming. Maximum roll angles in S2 showed no such relationship with TBF.

### 3.3 External and internal temperatures

Both tags recorded the ambient water temperatures experienced by the tagged sharks (Figure 2). Maximum water temperatures were 18.9 and 18.8°C for S1 and S2, respectively, and were recorded in the upper 2 m of the water column. S1 experienced a minimum temperature of 15.8°C at 46.6 m, with S2 experiencing a minimum temperature of 14.6°C at 209.2 m. S1 typically occupied warmer waters than S2 with a mean temperature of 17.9°C (±0.8 s.d.) compared to 16.4°C (±1.0 s.d.) recorded for S2.

Variation between internal (dorsal white muscle) and ambient water temperatures of S1 is presented in Table 3. Internal muscle temperatures of S1 ranged from a maximum temperature of 20.8°C to a minimum of 18.7°C (mean: 19.4°C ± 0.5 s.d.) (Figure 5c). Peak temperatures were recorded immediately post tagging, with temperatures decreasing to the minimum over a period of *c*. 9 h (Figure 5c). White muscle was on average 1.6°C (±0.8 s.d.) warmer than external water temperatures, albeit with variation throughout the tracking period from a maximum difference of 4.0°C to a minimum of 0.2°C. The mean difference between internal and external temperatures declined after release, from 2.6°C (±0.6 s.d.) in the 1 h immediately post release to 1.3°C (±0.7 s.d.) during routine sustained swimming after recovery (7 h at liberty) (Figure 5c). The internal muscle temperature during the single burst swimming event increased by 0.24 from 19.38°C (recorded just before the burst was initiated) to 19.62°C in 12.5 min (Figure 5c). Internal muscle temperatures were on average 3.5°C (±0.4 s.d.) higher than ambient water temperatures during burst swimming.

**TABLE 3:** Internal muscle temperatures and ambient water temperatures of shortfin mako shark (*Isurus oxyrinchus*) S1 across temporal periods of tracking

	Overall (mean ± 1 s.d.)	Post release (mean ± 1 s.d.)	Recovery period (mean ± 1 s.d.)	Routine swimming (mean ± 1 s.d.)	Burst swimming (mean ± 1 s.d.)
Internal muscle temperature (°C)	19.4 ± 0.5	20.5 ± 0.3	19.7 ± 0.2	19.0 ± 0.2	19.5 ± 0.1
Ambient water temperature (°C)	17.8 ± 0.8	17.9 ± 0.5	17.9 ± 0.8	17.7 ± 0.7	16.0 ± 0.3
Difference between internal and ambient temperature (°C)	1.6 ± 0.8	2.6 ± 0.6	1.8 ± 0.7	1.3 ± 0.7	3.5 ± 0.4

*Note*: Post release corresponds to the first hour of tracking post release. Recovery periods correspond to the period of 2–7 h where speed and field metabolic rate estimates are elevated above the mean. Routine swimming corresponds to periods of track after the recovery period except burst swimming. Burst swimming corresponds to the 12 min of internal muscle heating related to a burst swimming event.

### 3.4 Gliding behaviour

**FIGURE 6:**
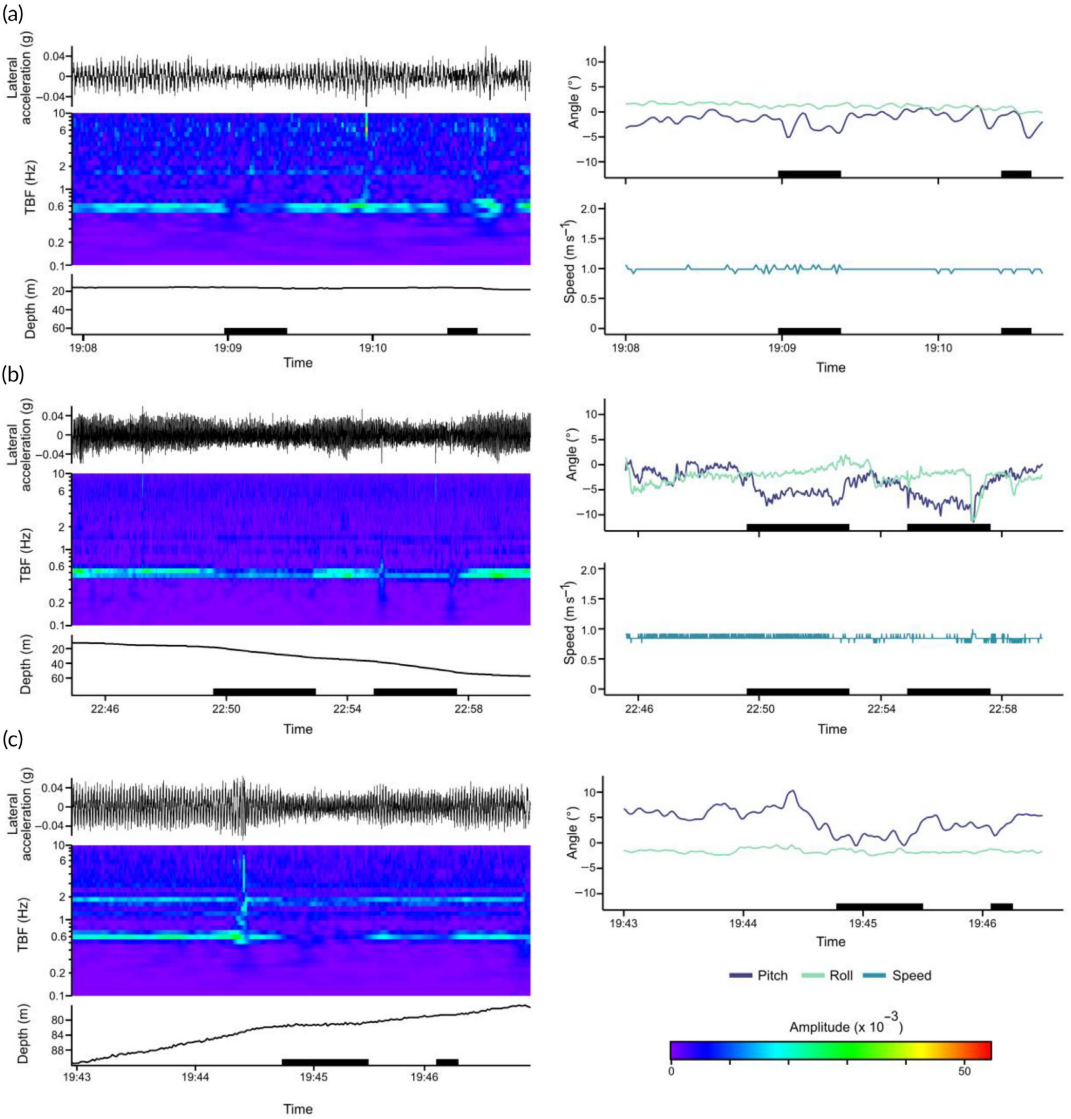
Examples of passive gliding behaviour and associated behavioural metrics across dive phases in shortfin mako sharks (*Isurus oxyrinchus*), where black lines above the spectrogram represent the dynamic lateral acceleration (*g*) recorded by accelerometers; spectrograms are coloured based on amplitude at a given frequency; black lines below the spectrograms represent depth (m); dark lines in the angle plot denote the pitch angle of the shark; and the lighter line represents the roll angle of the shark. (a) Example of short‐lived gliding during horizontal swimming from S1. (b) Example of successive glides during descents from S1 with increases in pitch angle associated with the cessation of tail beat. (c) Example of gliding during ascent, from S2, with associated decreases in pitch angle with the cessation of tail beat, NB. Speed was not directly measured for S2, so no speed data are available for gliding ascents. Black bars on the *x*‐axis denote periods of passive gliding

Gliding (zero TBF) was identified from the CWT outputs for both individuals (Figure 6), with 316 discrete gliding events totalling 4171 s for S1 (7.3% of total track time) and 401 events totalling 8485 s for S2 (6.5% of total track time). The longest durations of sustained gliding events were 201 and 414 s for S1 and S2, respectively. Gliding was most prevalent during descent phases comprising 23.4 and 9.9% of descent times in S1 and S2, respectively, compared to 2.3 and 5.3% of horizontal swimming (Figure 6a,b). Gliding during ascent phases was primarily observed in S2, with 4.3% of ascent times identified as being passive gliding (*e.g*., Figure 6c), whereas glides during ascents were rarely observed in shark S1 (0.2% of ascent times), with all instances less than 5 s duration.

Glide phases during horizontal swimming in S1 were typically short in duration, with a mean duration of 5.2 s (±4.5 s.d.), compared to gliding phases during descents with a mean duration of 16.7 s (±28.3 s.d.) (Figure 6a,b). A similar pattern was observed in S2, although horizontal glides were typically longer (mean: 18.4 s ± 35.2 s.d.) than for shark S1, but with less difference in duration when compared to gliding phases during descents (mean: 19.7 s ± 37.8 s.d.). Glides during ascents in S2 were shorter than those during descents or horizontal swimming, with a mean duration of 12.3 s (±14.7 s.d.) (Figure 6c). Measured speeds of S1 overall during gliding were unaffected by cessations in tail beat during both horizontal and descending glides (Figure 6a,b). Gliding phases during descents were typically steeper than powered descents in S1 but were unchanged in S2. Gliding phases during ascents were generally shallow, with sharks assuming a near‐horizontal body position before resuming a steeper, powered ascent, with a commensurate reduction in ascent rate (Figure 6b,c).

**FIGURE 7:**
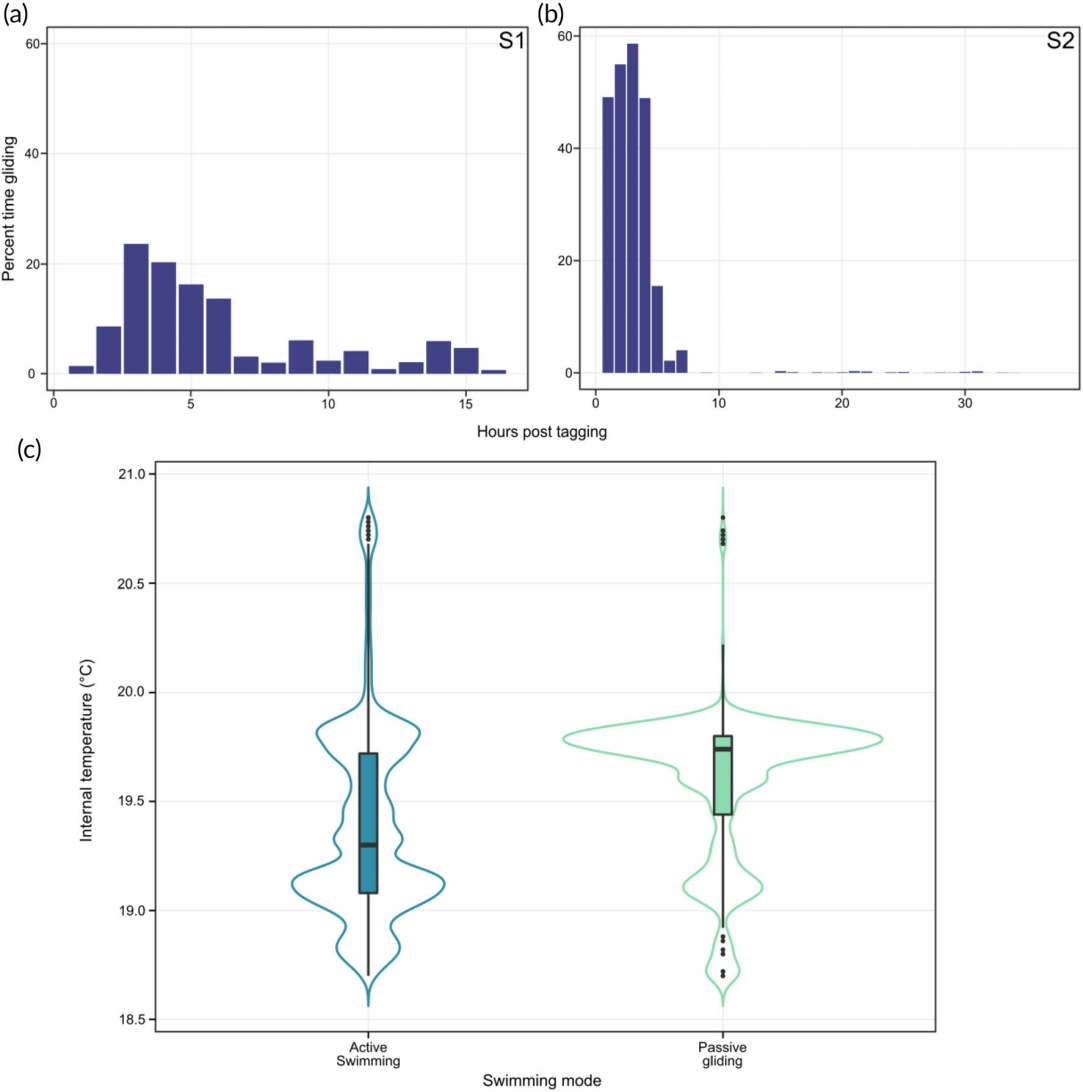
Summary of passive gliding events of tagged shortfin mako sharks. (a) Frequency of passive gliding during each hour of tracking post release of shark S1. (b) Frequency of passive gliding during each hour of tracking post release of shark S2. (c) Internal temperature of S1 during active swimming and passive gliding; the violin plot shows the kernel probability density; internal box plots display the median and interquartile ranges

Both sharks exhibited higher frequencies of gliding events within the first 5 h post tagging, with the peak in percentage time spent gliding in both sharks occurring within the third hour after release (Figure 7a,b). Gliding was more frequently observed in shark S2 within the initial 5 h period, with 58.6% gliding before declining to near zero after hour 7 (<0.5% of time spent gliding) (Figure 7b). Time spent gliding by S1 also increased in the first 3 h, but with a maximum of 23.6% of time spent gliding in hour 3. Gliding behaviours were observed more frequently in S1 than in S2 after hour 7, with an average of 5% of each hour spent gliding (Figure 7a,b). After hour 7 almost all observed glides occurred during descending phases, with glides during horizontal and ascending phases declining to near zero. S1's white muscle temperatures were higher during passive gliding phases than during powered swimming, with gliding phases being most prevalent when white muscle temperatures were above 19.5°C (Figure 7c).

### 3.5 Field metabolic rates

Speed‐based estimations of metabolic rate yielded a mean FMR of 198.6 mg O_2_ kg^−1^ h^−1^ (±48.3 s.d.)^−1^ for S1. The RMR, defined as the mean metabolic rate for those speed data points classified as cruising speeds, was 195.9 mg O_2_ kg^−1^ h^−1^ (±38.0 s.d.). FMR estimates were lower for S2, with a mean of 168.8 mg O_2_ kg^−1^ h^−1^ (±29.1 s.d.) and an RMR of 179.7 mg O_2_ kg^−1^ h^−1^ (±13.9 s.d.). Both sharks showed considerable variation in metabolic rate in the 7 h after release. Shark S1 displayed elevated metabolic rates within the first 2–3 h of deployment, with the 60 min rolling mean being up to 30% higher than the overall mean for the entire track. Metabolic rates then declined sharply, before stabilising *c*. 7 h post tagging. S2 showed a general reduction in metabolic output immediately post release, with the 60 min rolling mean 36% lower than the overall mean for the entire tracking period. Metabolic rates again stabilised after *c*. 7 h into S2 deployment.

## 4 DISCUSSION

Recent advances in bio‐logging technologies have rapidly increased our ability to understand the activity patterns and ecophysiology of large pelagic animals. There has been a burgeoning use of accelerometers and speed sensors to explore the swimming kinematics and biomechanical drivers of movement in a range of large fish species (Gleiss *et al*., 2019; Logan *et al*., 2023; Watanabe *et al*., 2019b). Several studies have explored the broad‐scale movement patterns of mako sharks using archival and satellite positioning tags (Francis *et al*., 2019; Queiroz *et al*., 2016; Vaudo *et al*., 2017); nonetheless, this study represents the first use of a fixed bio‐logging package capable of recording swimming speeds, fine‐scale activity and thermal biology simultaneously in this species. These results support the understanding of the mako shark as one of the fastest and most energetically active sharks, capable of high swimming speeds and periods of elevated energy expenditure, similar to other endothermic species such as white sharks (Anderson *et al*., 2022; Semmens *et al*., 2013). However, the present study obtained speeds for only two individuals. Although studies with low sample sizes are capable of offering invaluable insight into aspects of animal behaviour and ecology, they preclude the ability to capture the full range of variability that may exist within a population or species (Sequeira *et al*., 2019). Therefore, detailed measurements such as those shown here but for a greater number of makos are required to more fully understand its swimming behaviours and energetics.

### 4.1 Swimming speeds

The sustained cruising speeds of pelagic fishes play a crucial role in their ecology and life history, as individuals should find an optimal balance between efficiency of transport, *i.e*., energy expenditure, and efficiency of foraging, *i.e*., the rate of prey encounters and resultant captures (Watanabe *et al*., 2019b; Weihs, 1973). Comparatively, the cruising speed of shortfin mako is similar to that of other regionally endothermic fish (*c*. 0.7–1.5 m s^−1^) (Table 4). The measured cruising speed recorded in the present study matches that of an unpublished direct measurement of the cruising speed of a mako shark (Carey, unpubl. data; cited in Block *et al*., 1992) (Table 4) but is faster than that recently measured from makos tagged with towed bio‐logging packages (Saraiva *et al*., 2023) (Table 4). The makos tagged by Saraiva *et al*. were smaller than those tagged in this study (*c*. 1.50 *vs*. 2.00 m), potentially explaining the lower recorded swimming speeds. Ontogenetic shifts in swimming speeds are suggested for white sharks (Anderson *et al*., 2022); nonetheless, this is yet to be explored for mako sharks. Differences in tag attachment method (fixed, this study; towed, Saraiva *et al*., 2023) may also be a factor in the variation in speed measurements found. Mako cruise swimming speeds recorded in this study were similar to those previously directly measured for white shark (Watanabe *et al*., 2019b) but lower than those for bluefin tuna (*Thunnus thynnus*) (Gleiss *et al*., 2019) and sailfish (*Istiophorus platypterus*) (Marras *et al*., 2015). In contrast, the cruising swimming speed of a Pacific bluefin tuna (*Thunnus orientalis*) was lower than that of tracked makos; however, the tuna measurements were for a small (0.5 m long) individual within a sea cage, so it may not accurately represent the true cruising speeds of wild adult specimens (Komeyama *et al*., 2011).

**TABLE 4:** Reported sustained and burst speeds of large pelagic fishes directly measured using animal‐borne speedometers

Species	Method	Total body length (m)	Sustained speed (m s^−1^)	Burst speed (m s^−1^)	Burst speed duration (s)	Depth (m)	Swimming direction	Reference
Shortfin mako shark *Isurus oxyrinchus*	Speedometer (F)	2.0	0.90	5.02	14	15	Descending	This study
	Speedometer (NR^a^)	1.8	0.90	1.50	–	–	–	Carey, unpublished; cited in Block *et al*. (1992)
	Speedometer (T, 1.0 m)	1.4–1.6	0.50–0.62	3.69	–	–	–	Saraiva *et al*. (2023)
White shark *Carcharodon carcharias*	Speedometer (F)	3.8	–	6.70	19	0	Ascending	Watanabe *et al*. (2019a)
	Speedometer (F)	2.9–4.3	0.90	–	–	–	–	Watanabe *et al*. (2019b)
Oceanic whitetip *Carcharhinus longimanus*	Speedometer (F)	2.2–2.85	0.6–0.7	4.6	–	–	–	Papastamatiou *et al*. (2018)
Blue shark *Prionace glauca*	Speedometer (F)	1.6–1.7	0.4	2.0	–	–	–	Watanabe *et al*. (2021)
Bluefin tuna *Thunnus thynnus*	Speedometer (F)	2.4^b^	1.5	2.50	–	0–40	Horizontal	Gleiss *et al*. (2019)
Pacific bluefin *Thunnus orientalis*	Speedometer (T, NR)	0.5	0.71	3.60	–	Cage	Horizontal	Komeyama *et al*. (2011)
Blue marlin *Makaira nigricans*	Speedometer (T, 0.35 m)	1.8	0.5–1.0	2.25	–	60	Descending	Block *et al*. (1992)
Sailfish *Istiophorus platypterus*	Accelerometer (F)^c^	2.0–2.2	1.95	7.02	–	0–5	Horizontal	Marras *et al*. (2015)
	High‐speed video^c^	2.0–2.2	2.30	5.23	–			
	Speedometer (F)	1.85	0.56–1.30	3.1	–		Ascending	Logan *et al*. (2023)

*Note*: All values are given to the same decimal places as reported in original studies. Speedometers were either fixed to the fin or body (F) or towed (T) by the fish on a tether (length in metres). NR denotes speedometer attachment method or towed tether length was not reported in the literature cited.

^a^ Speedometer was probably towed on a 0.35 m tether (*cf*. Block *et al*., 1992).

^b^ Curved fork length.

^c^ For both methods in this study, tail‐beat frequencies (TBF) were used to estimate swimming speeds during burst and cruising.

Large predatory fish are often categorised as either “energy speculators” – exhibiting a life history of higher swimming speeds and maximising energy acquisition at the expense of high energy demands (*e.g*., tunas) (Dickson, 1995) – or “energy hedgers,” where swimming speeds and energetic costs are minimised and where energy acquisition rates may be lower (Papastamatiou *et al*., 2018). The majority of ectothermic pelagic shark species typically fall into the latter category, including oceanic whitetip (*Carcharhinus longimanus*) and blue shark (*Prionace glauca*), which typically have slower cruising speeds than found for mako in this study (Table 4) (Papastamatiou *et al*., 2018; Watanabe *et al*., 2021). The higher cruising speeds of the mako suggest that they are “energy speculators,” with the extensive convergent physiological and morphological evolution between tunas and lamnids, resulting in adaptations that enable mako sharks to sustain such high swimming speeds (Bernal, Dickson, *et al*., 2001). Higher sustained swimming speeds of endothermic fishes are likely to increase the chances of prey encounters, or result in higher success rates due to faster pursuit speeds (Harding *et al*., 2021), while also facilitating large‐scale migrations (Watanabe *et al*., 2015). The elevated cruising speeds may also enable the mako to undertake ocean‐basin scale movements of several thousand kilometres annually and exploit a wider geographic range than would be possible for the majority of slower swimming species (Nasby‐Lucas *et al*., 2019; Watanabe *et al*., 2015). Nevertheless, the high energetic cost of such a strategy will necessitate either frequent feeding or the targeting of large, energy‐dense prey (Papastamatiou *et al*., 2018) that may also swim fast.

The measured maximum (burst) speed of 5.02 m s^−1^ was faster than that previously recorded for a single mako shark by F. G. Carey (1.50 m s^−1^; Carey, unpubl. data, cited in Block *et al*., 1992). This high burst speed by S1, coupled with the rapid changes in body orientation, culminating in a tight turn before deceleration by gliding, suggests it was pursuing prey. This behaviour is likely linked to the extensive network of seamounts within the area which are known to aggregate migratory predators such as mako (Morato *et al*., 2008; Morato *et al*., 2010). Probable foraging in the vicinity of seamounts supports previous hypotheses that seamounts aggregate prey creating a “feeding station” for large pelagic predators (Morato *et al*., 2008) and demonstrates the value of direct speed measurement coupled with accelerometry to reveal key ecological processes during short‐term deployments.

The maximum speed in the current study was consistent with those directly measured from free‐swimming sharks and other large pelagic fishes (Table 4), with the only higher speeds being those recorded for sailfish and white sharks, that were also measured during predation events (Marras *et al*., 2015; Watanabe *et al*., 2019a). The measured maximum was, however, below the maximum estimate for this species. Carey and Teal (1969) calculated the escape velocity of breaching makos after hooking by sport fishers to be greater than 9.8 m s^−1^, with theoretical speed estimates as high as 20 m s^−1^ (Motta *et al*., 2012). Such differences between *in situ* speed measurements and estimations based on other methods are common across species. For example, the maximum speeds of sailfish have been estimated at 30 m s^−1^ based on fishers' estimations of escape speeds of hooked individuals (Lane, 1941; Svendsen *et al*., 2016), whereas the maximum speed directly measured for this species is an order of magnitude lower at “only” 7.05 m s^−1^ (Marras *et al*., 2015).

Given the apparent disparity between indirectly estimated speeds (*e.g*., after hooking by rod‐and‐line) and the burst speeds directly recorded for sharks and other fishes under natural conditions, it is probable that the maximum speed recorded here may underestimate the maximum speed achievable by shortfin mako. The differences between direct speed measurements and those estimated during hooking of fish during angling probably more accurately reflect the difference between speeds attained during hunting prey and those when attempting to escape a predator. Models concerning the relationship between activity level and mortality predict that as time at maximum activity increases, so do the chances of death, necessitating animals to remain within metabolic limits (Priede, 1977). One occasion where such considerations may differ is during predator escape, where the risk of predation mortality outweighs the risks of mortality from elevated activity levels (Priede, 1977). This may in part explain the presence of exceptionally high speeds observed in response to fisheries capture in large pelagic fishes, such as the mako, where under normal conditions prey is selected that can be pursued within energetic limits resulting in lower maximum speeds. Recent research into the hunting behaviours of sailfish also suggests that large pursuit predators will rarely reach their maximum speeds in the pursuit of prey, with prey items setting the maximum speed of the pursuit (Logan *et al*., 2023). As pursuit predators must be able to outpace prey or at least maintain pace to the point of prey exhaustion for predations to be successful, it is doubtful that predators will select prey they are unable to pursue (Guinet *et al*., 2007). It is also possible that the presence of fin‐mounted tags and their associated drag may limit the maximum speed a tagged animal can attain (Kay *et al*., 2019).

### 4.2 Swimming kinematics

Mako TBF and speed exhibited a linear relationship similar to other shark species (Nakamura *et al*., 2011; Watanabe *et al*., 2019a). Sustained periods of elevated TBF were infrequent; for example, higher TBF during burst swimming was sustained for 14 s at a mean of 2.97 Hz. Mean TBF measured for mako was similar to other large shark species such as tiger shark (*Galeocerdo cuvier*) and white shark, which typically maintain a TBF of *c*. 0.4–0.5 Hz (Nakamura *et al*., 2011; Semmens *et al*., 2019). Increases in mako TBF were similar in both frequency and duration to white shark breaching events where TBF increases from 0.39 to 2.50 Hz over brief periods of 7–16 s (Semmens *et al*., 2019). The mean TBF was lower than bluefin tuna, which maintains a regular swimming TBF of *c*. 0.80–0.95 Hz, and was also able to maintain TBFs of 2 Hz for periods of several minutes (Gleiss *et al*., 2019). Sailfish TBF has also been recorded above that of the mako (1.54–2.02 Hz), which helps explain bluefin tuna and sailfish ability to cruise at higher speeds over prolonged periods (Table 4) (Gleiss *et al*., 2019; Marras *et al*., 2015). Maximum TBF was also higher in sailfish, with TBF during burst swimming between 4.15 and 6.15 Hz supporting speeds of 5.23–7.02 m s^−1^ (Marras *et al*., 2015).

For mako, TBF was lower during descents than during other dive phases in both individuals, which was largely due to the higher frequency of powerless, passive gliding phases. Passive gliding was first proposed by Weihs (1973) as a mode by which negatively buoyant fish may reduce energetic transport costs and has been observed across a range of pelagic shark and scombrid species (Gleiss *et al*., 2019; Watanabe *et al*., 2019b; Watanabe *et al*., 2021). Energy expenditure estimates for white shark suggest energy savings of up to 29% during deep gliding descents compared to powered surface swimming (Watanabe *et al*., 2019b). Mako gliding in the present study was most prevalent during the first 7 h of both tracks with a dramatic reduction in frequency after this time, which suggests gliding as a behavioural response to capture that is aimed at reducing power output and metabolic heat production. In addition to alterations in gliding, both sharks tagged in this study undertook initial dives to *c*. 50 m immediately post release, before resuming regular vertical movements and activity after *c*. 90 min, which may have been related to reoxygenation or thermoregulation (Holts & Bedford, 1993), or as an escape response from surface capture by diving. Measured swimming speeds were also elevated conforming to the typical swimming response of ram ventilators after capture (Iosilevskii *et al*., 2022). Mako speed increases were lower and shorter in duration than those recorded for the majority of other species however (Iosilevskii *et al*., 2022), possibly linked to the ability of mako sharks to rapidly metabolise anaerobic by‐products after periods of intense energy expenditure (Bernal *et al*., 2012).

When only periods of powered swimming were considered, there was no difference in TBF between ascents, descents or horizontal swimming in S1 similar to bluefin tuna (Gleiss *et al*., 2019). This effect may be driven by a biomechanical need for fish to sustain a regular TBF through dive phases as a means of maintaining propulsive efficiency due to the hydrodynamic properties of the caudal fin (Taylor *et al*., 2003). S2 in contrast showed an increase in TBF during ascents compared to descents and horizontal swimming. Tuna have been shown to increase TBF in ascents from deep dives when compared to shallow dives (Gleiss *et al*., 2019), suggesting the deeper depth distribution of S2 may account for this alteration in TBF, although the absence of repetitive shallow dives in this individual meant it was not possible to test this relationship further.

### 4.3 Energy conservation

Dorsal white muscle temperatures of S1 were typically 1.6°C higher than ambient, within the range measured by Bernal, Sepulveda, and Graham (2001) (0.3–3°C above ambient), but lower than the 4°C difference reported from free‐swimming makos by Carey *et al*. (1985). The body temperatures recorded by Carey *et al*. were from large, decked specimens, and the stress of capture and handling very likely elevated internal temperatures (Bernal, Sepulveda, & Graham, 2001; Carey *et al*., 1985). In the present study a similar difference in body to water temperature to that obtained by Carey *et al*. (1985) was found after burst swimming, although this can in large part be attributed to mako S1 diving into colder water as body temperature increased. Reduction in gliding frequency matched the decrease in white muscle temperatures of S1 during the first 7 h of tracking. Results from laboratory‐based experiments suggest that although makos are capable of a substantial degree of physiological control of internal temperatures, cooling rates of muscle are significantly slower than warming rates (Bernal, Sepulveda, & Graham, 2001). Gliding may therefore aid in enhancing transport efficiency but also serve as a means of energy recovery after periods of energy expenditure (*e.g*., post tagging or burst swimming) and in cooling body temperatures to optimal ranges by reducing red muscle activity and associated heat generation.

After apparent recovery from capture and tagging, non‐powered glides accounted for 11.54% of descents in S1, a similar level to white sharks undertaking similar shallow dives to less than 50 m (8%) (Watanabe *et al*., 2019b), but which was only 0.18% in S2. Individual differences in gliding frequency have been observed in other species, with no glides recorded during tracking of individual white sharks and tiger sharks (Andrzejaczek *et al*., 2020; Watanabe *et al*., 2019b), with such individual variation resulting from the relationship between swimming speeds and biology or morphology. Sharks with high liver lipid contents may negate the need for gliding as their near‐neutral buoyancy may allow for the reduction in swimming speeds while still maintaining hydrodynamic lift from the fins (Watanabe *et al*., 2019b). Conversely, the efficiency gains of passive gliding will also decrease with increasing swimming speeds due to the increase in incurred hydrodynamic drag, leading to an increased time spent actively swimming (Weihs, 1973). Given the similar body sizes and swimming speeds of makos in this study, it is unlikely that differences in buoyancy‐ or speed‐related drag will explain differences in gliding. S2 exhibited no periods of burst swimming and spent extended periods at deeper depths with lower ambient temperatures than S1. If gliding is linked to reducing metabolic heat production as a result of periods of elevated higher power outputs, such as during capture or burst swimming, the between‐individual differences could be accounted for by variations in activity and thermal environment. Further deployments of motion‐sensitive bio‐loggers and accompanying physiological data will be required to test the role that internal temperature plays in governing the swimming kinematics of mako sharks.

### 4.4 Energetics

Reported metabolic rates of large sharks are largely estimated FMRs due to the logistical complexity of experimentally measuring oxygen consumption in such species (Payne *et al*., 2015). To date, FMRs have been estimated for only a handful of larger elasmobranch species, including Greenland shark (*Somniosus microcephalus*) (Ste‐Marie *et al*., 2022), bull shark (*Carcharhinus leucas*) (Lear *et al*., 2020), basking shark (Sims, 2000) and white shark (Anderson *et al*., 2022; Semmens *et al*., 2013). Mako metabolic rates were higher than FMRs estimated for large cold‐water species, *e.g*., the Greenland shark (25.8 mg O_2_ kg^−1^ h^−1^) (Ste‐Marie *et al*., 2022) and the filter‐feeding basking shark (80.7 mg O_2_ kg^−1^ h^−1^) (Sims, 2000). In contrast, the estimated FMR of mako in the present study was 50.3% less than that of white sharks of similar sizes (Anderson *et al*., 2022) but which can be most likely accounted for by the methodological differences in estimation methods used between these studies. Estimating FMR requires several input parameters that may be incompletely known or only partially applicable. The SMR of juvenile mako was utilised in FMR calculations in this study, whereas previous white shark studies used a metabolic rate measured for YOY white sharks that were able to swim within a restricted space (Anderson *et al*., 2022; Ezcurra *et al*., 2012; Semmens *et al*., 2013), resulting in an estimate more than double that of the mako SMR (Ezcurra *et al*., 2012; Sepulveda *et al*., 2007). The species‐specific SMR has the advantage of removing any influence of activity level from experimentally derived metabolic rates on estimated FMR calculations. For example, the RMR of juvenile mako sharks was measured at 2.5 times that of the SMR, which would have a proportional impact on FMR estimates (Sepulveda *et al*., 2007). Furthermore, mako estimates here include the use of a scaling exponent to adjust the SMR estimates of juvenile sharks up to the size of tagged sharks. The choice of the scaling exponent can have substantial impact on the estimates of metabolic rate returned for an individual, especially when scaling from very small to large body sizes (Lawson *et al*., 2019). The conservative scaling exponent of 0.79 from Payne *et al*. (2015) was used here. Nonetheless, using a scaling exponent of 0.89 (Nagy, 2005) would result in a metabolic rate estimate 28% higher than currently estimated (Supporting Information Figure S3; see Data S1: Sensitivity analysis). Therefore, comparing FMR between species is problematic when methods vary. Further advances in respirometry, in particular seagoing “megaflumes” capable of directly measuring metabolic rates of large sharks, will help minimise the discrepancies between estimation methods (Payne *et al*., 2015). Nonetheless, these FMR estimates support the notion of the mako shark as a highly energetically active species.

The method for estimating FMR applied here uses oxygen consumption as a proxy for metabolic rate and thus can be used to estimate only aerobic metabolism. In mako, and other lamnids, rapid and short‐lived accelerations in swimming speeds, such as during predations, are largely supported by the white muscle (Bernal, Dickson, *et al*., 2001). Such activities are likely to necessitate engagement of anaerobic pathways, with the high concentrations of anaerobic metabolic enzymes within mako shark white muscle facilitating the high energy required for burst swimming, in addition to promoting the rapid removal of anaerobic by‐products from muscle tissues (Bernal *et al*., 2003; Bernal, Dickson, et al., 2001). Consequently, the method used here to estimate FMR is unlikely to be appropriate to estimate metabolic rate during such behaviours. The rapid increase in muscle temperatures recorded as a result of burst swimming suggests that high‐speed swimming is energetically expensive and may necessitate the selection of large, energy‐dense prey by shortfin mako. Stomach contents of mako sharks caught in beach protection methods in South Africa support this hypothesis, with prey items typically falling within the range of 23%–35% of the total body length of the shark predator (Cliff *et al*., 1990). The daily costs of pursuit of multiple, fast‐swimming prey compared with selective pursuit of a single large prey may explain the low frequency of such events during tracking in this study, with only one burst speed >5 m s^−1^ recorded in >50 h of tracking.

### 4.5 Energy expenditure and climate change

The results presented here have implications in future assessments of mako shark responses to climate‐driven changes in the ocean environment. Given the mako's feeding ecology of predating on large, fast‐moving teleosts (Wood *et al*., 2009), the ability to successfully pursue prey at high speeds is a key component of an individual's fitness. Therefore, any alteration in the capacity of mako sharks to undertake metabolically demanding behaviours will impact energy acquisition rates and predator avoidance, with consequences at both the individual and population levels. The impact of climate changes on shark swimming capabilities is largely unknown, especially for large endothermic species. Warming temperatures enhance burst swimming performance in some fish species (Vilmar & Di Santo, 2022). However, temperature‐induced increases in SMR may reduce aerobic scope and the capacity to undertake high‐energy behaviours (Claireaux & Chabot, 2016). Laboratory studies have demonstrated mako sharks' ability to regulate body temperatures in waters of at least 26°C, although if the observations of passive gliding increasing with higher body temperatures in the current study are general across this species, alterations in swimming patterns of makos may occur as a result of increasing temperatures. Recent studies suggest that endothermy in marine fish is not linked to an expansion of thermal niche but that it increases power output (Harding *et al*., 2021). Mako sharks are likely to be vulnerable to the effects of ocean warming, which may increase standard metabolic costs and reduce aerobic scope. In addition, high‐oxygen‐demand fishes such as makos are likely to be affected by expansions in low DO environments associated with ocean deoxygenation (Vedor *et al*., 2021). Reductions in ambient oxygen concentrations increase recovery time from high‐energy events such as capture or burst swimming in marine fish, with low DO limiting capacity to metabolise anaerobic by‐products (Shultz *et al*., 2011). This has the potential to limit the frequency of burst swimming and the aerobic habitat where hunting may occur. DO concentrations and ocean deoxygenation also constrain pelagic shark diving behaviours, reducing habitable space (Vedor *et al*., 2021). This habitat compression, coupled with the requirement to recover any incurred oxygen debts in shallow, oxygenated mixed layers, may also increase the risk of capture by fisheries above or within oxygen‐depleted environments (Vedor *et al*., 2021). A focus on ecophysiological bio‐logging of pelagic fishes will enable the effects of climate‐driven changes on threatened makos to be quantified.

## 5 CONCLUSIONS

This study was the first deployment of fixed, fin‐mounted tri‐axial accelerometers, speed and internal temperature sensors on free‐swimming mako sharks in their natural environment and provides baseline measurements of swimming speeds, TBF and FMR estimates. Mako cruising speeds are shown to be elevated above that of large ectothermic sharks of similar body size but similar to other endothermic sharks. The burst swimming speed measured was among the highest yet reported for large, pelagic fishes, but such high speeds appear relatively rare, suggesting it is energetically expensive. The distribution of periods of powerless glide swimming suggests its role in energy recovery post‐exercise, rather than just as a means of increasing transport efficiency, and with a potential further role in thermoregulation by reducing post‐capture metabolic heat production. Collectively, the results highlight the potential vulnerability of this endangered species to the effects of anthropogenic climate change such as increased warming and deoxygenation with regard to its high‐speed performance.

## AUTHOR CONTRIBUTIONS

Nuno Queiroz and David W. Sims conceived the research. Bruno Loureiro developed software and hardware tools. Matt J. Waller, Nuno Queiroz, Ivo da Costa, Freya C. Womersley, Jorge Fontes, Pedro Afonso, Bruno C. L. Macena, Nicolas E. Humphries and David W. Sims performed fieldwork and data collection. Matt J. Waller analysed the data and prepared the manuscript. Nuno Queiroz, Ivo da Costa, Tiago Cidade, Freya C. Womersley, Alexandra Loveridge and David W. Sims provided additional analysis. All authors contributed to subsequent drafts.

## FUNDING INFORMATION

Fundacao para a Ciencia e a Tecnologia (FCT) PTDC/BIA/28855/2017, COMPETE POCI‐01‐0145‐FEDER‐028855 and CEECIND/02857/2018 (all Nuno Queiroz); MARINFO–NORTE‐01‐0145‐FEDER‐000031 [funded by Norte Portugal Regional Operational Program (NORTE2020) under the PORTUGAL 2020 Partnership Agreement, through the European Regional Development Fund–ERDF] (Nuno Queiroz); UK Natural Environment Research Council (NERC) Discovery Science (NE/R00997/X/1) (David W. Sims); European Research Council (ERC) under the European Union's Horizon 2020 research and innovation programme (ERC‐AdG‐2019 883583 OCEAN DEOXYFISH) (David W. Sims); and Marine Biological Association (David W. Sims).

## Supporting information


**DATA S1.** Supporting information.
