# Supplementary material for: Direct measurement of cruising and burst swimming speeds of the shortfin mako shark (Isurus oxyrinchus) with estimates of field metabolic rate
Source: J Fish Biol. 2023 Jul 4;103(5):864–83. doi: 10.1111/jfb.15475 (PMC10952363; doi:10.1111/jfb.15475)
Supplement: Supplementary file 1 — DATA S1. Supporting information. [file JFB-103-864-s001.docx]

**Supplementary Information**

## **Tag Calibration**

Calibration of the ORI1300 3MPD3GT was conducted within the confines of the Port of Leixões, Porto, Portugal (Supplementary Figure 1). A linear model of logger recorded speeds against actual boat speed was used to correct speeds recorded during deployment (Supplementary Figure 2).


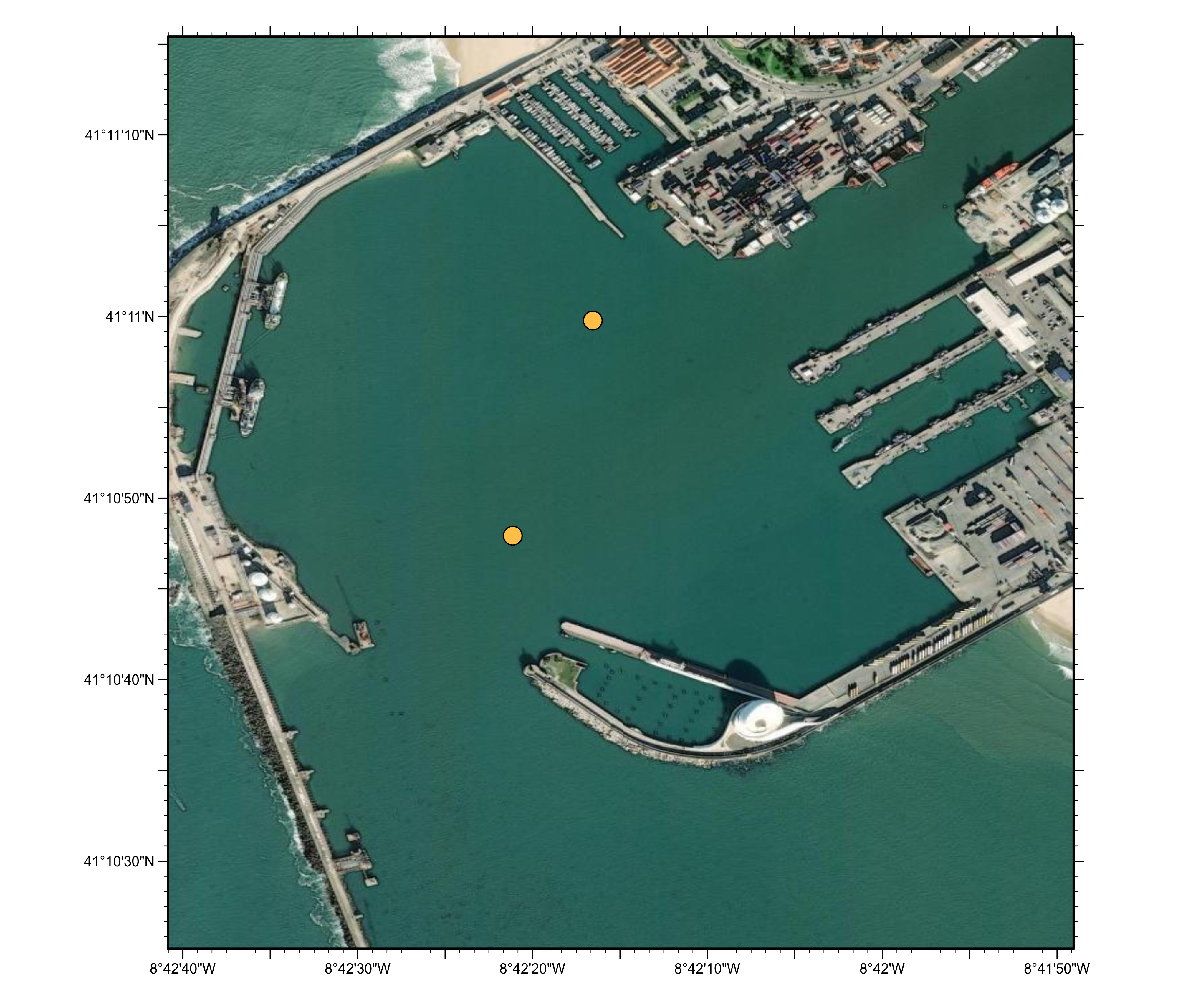


Supplementary Figure 1: Satellite image of the Port of Leixões, Porto, Portugal. Orange circles represent the start and end points of the calibration runs. Map produced in Arc GIS Pro 2.7.7 (ESRI, Redlands CA).


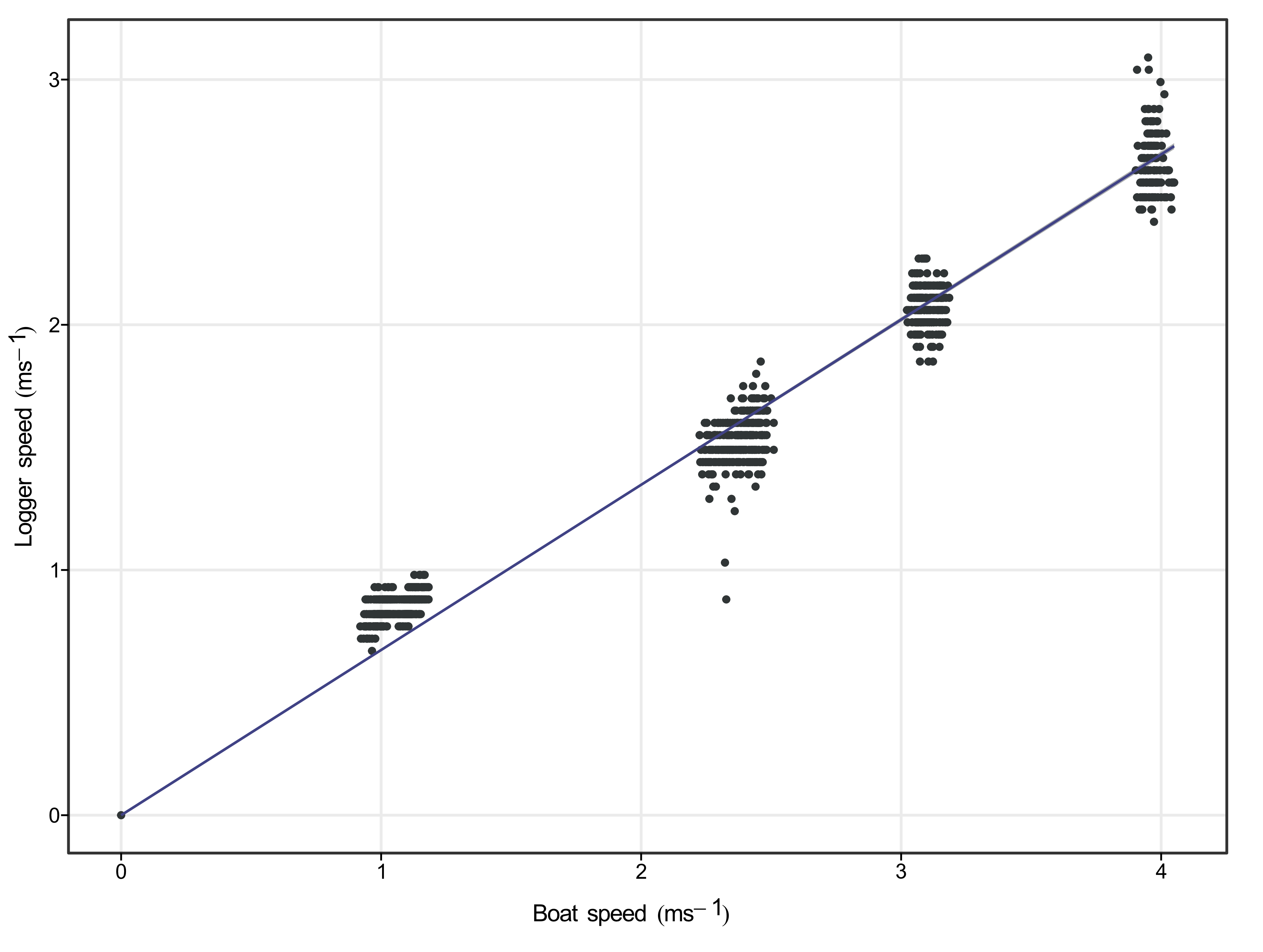


Supplementary Figure 2: Results of speed calibration of speed sensor. Circles show speed readings of logger at each test speed. Line shows the results of linear model (lm) used to correct speeds recorded during deployments, shaded area shows SE of this model. The fitted lm: corrected speed = 1.48 * original speed + 0.

## **Weight Estimation**

Supplementary Table 1: Total length and fork length of tagged shortfin mako sharks, lengths are given in cm.

| ID | Total length (TL) | Fork length (FL) |
| --- | --- | --- |
| S1 | 197 | 186 |
| S2 | 199 | 186 |

Supplementary Table 2: Length-Weight calculations for shortfin mako from the Atlantic. TL = total length, FL = fork length

| Reference | Equation |
| --- | --- |
| Alahyene and et al. (2021) | W = (7 x 10^-5^)TL^2.6092^ |
| Kohler et al., (1996) | W = (5.2432 x 10^-6^)FL^3.1407^ |
| Mejuto et al., (2007) | W = (4.67098 x 10^-6^)FL^3.16457^ |

{Alahyene, 2021 #345}

Supplementary Table 3: Weight estimates for tagged shortfin mako sharks, all weight estimates are given in kg

| ID | Weight 1 (Alaheyne et al. 2021) | Weight 2 (Kohler et al. 1996 | Weight 3 (Mejuto et al. 2008) | Mean estimate |
| --- | --- | --- | --- | --- |
| S1 | 67.892 | 70.381 | 71.029 | 69.798 |
| S2 | 69.705 | 70.381 | 71.029 | 70.372 |

## **Sensitivity Analysis**

The effect of differing mass scaling exponents and Q_10_ values on FMR estimations were tested using both theoretical data and real world data from mako shark S1. The range of scaling exponents tested were chosen after Payne et al., (2015). The difference in FMR estimates from speed based estimations is constant between scaling exponents, for example FMR estimates using an exponent of 0.79 are always 1.34 times those estimated using an exponent of 0.67 (Figure 3A). When applied to the real speed data directly measured from S1 mean FMR was 148.3 mg O_2_ kg^-1^ h^-1^ (± 36.1 S.D.) using an exponent of 0.67, 198.7 mg O_2_ kg^-1^ h^-1^ (± 47.3 S.D.) using an exponent of 0.79 and 253.5 mg O_2_ kg^-1^ h^-1^ (± 61.6 S.D.) using an exponent of 0.89 (Figure 4B). We tested a range of Q_10_ values reported in published literature, 2.42 a value from nurse shark (*Ginglymostoma cirratum*) (Whitney et al., 2016), 1.65 a value reported for fish (White et al., 2006) and 1.34 from scalloped hammerhead shark (*Sphyrna lewini*) (Lowe 2001). he effect of no temperature correction on FMR estimates was also tested. A higher Q10 value will give a higher variability between FMR estimates at different ambient water temperatures assuming a constant swimming speed (Figure 3C). Though variation in FMR will result from differing Q10 values little impact was found on our data with FMR estimates ranging from 197.0 mg O_2_ kg^-1^ h^-1^ (± 48.6 S.D.) at a Q10 of 2.42 to 199.6 mg O_2_ kg^-1^ h^-1^ (± 48.7 S.D.) with no correction for ambient temperature (Figure 3D).


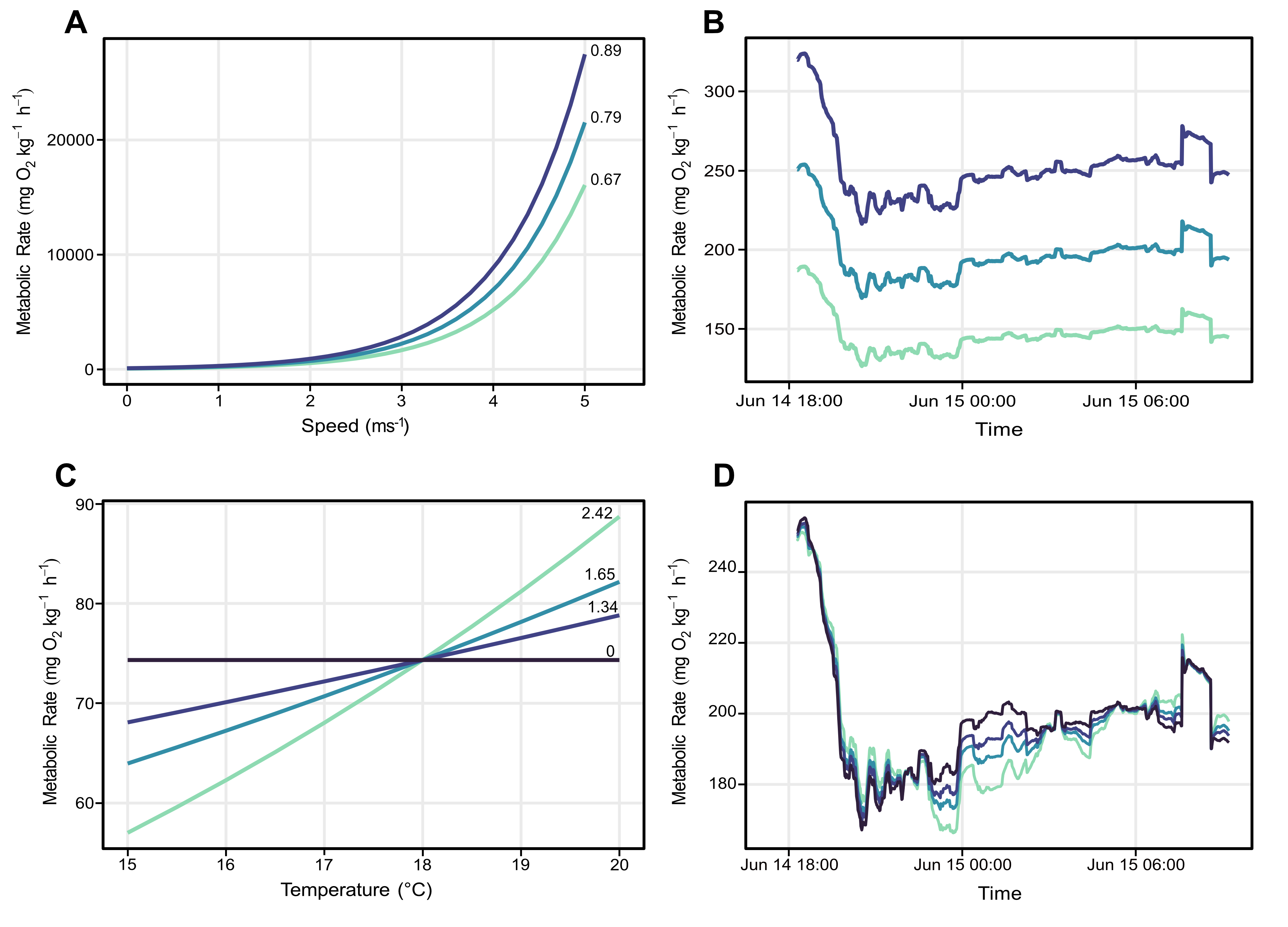


Supplementary Figure 3: Effects of scaling exponents and Q_10_ values on FMR estimates from shortfin mako. Panel A**:** FMR estimates at different speeds using three different scaling exponents at a constant temperature of 18^o^C, numbers show the scaling exponents used in sensitivity analyses. Panel B: the effect of the same exponents on FMR estimates from shark S1 with FMR estimates smoothed across an hour. Panel C**:** the effect of differing Q10s on SMR at a given temperature at a constant swimming speed of 1 body length per second, numbers show the Q_10_ values used in sensitivity analyses. Panel D: the effect of the same Q_10_ values on FMR estimates from shark S1 smoothed across an hour.

## **References**

Alahyene, Jihade, and et al. 2021. The Growth Parameters of the Mako Shark Isurus Oxyrinchus (Rafinesque, 1810) in the Moroccan Central Atlantic Coast, *Egyptian Journal of Aquatic Biology and Fisheries*, 25: 119-36.

Kohler, N. E., Casey, J. G., and Turner, P. A. 1996. Length-length and length-weight relationships for 13 shark species from the western north Atlantic. In *NOAA technical memorandum NMFS-NE ; 110*.

Lowe, C. G. 2001. Metabolic rates of juvenile scalloped hammerhead sharks (Sphyrna lewini), *Marine Biology*, 139: 447-53.

Mejuto, J., Ramos-Cartelle, A., Quintans, M., Gonzalez, F., and Carroceda, A. 2007. Length-weight relationships and morphometric conversion factors between weights for the blue shark (*Prionace glauca*) and shortfin mako (*Isurus oxyrinchus*) caught by the Spanish surface longline flee in the Atlantic ocen. In *ICCAT SCRS/2007/079*, 13.

Payne, N. L., Snelling, E. P., Fitzpatrick, R., Seymour, J., Courtney, R., Barnett, A., Watanabe, Y. Y., Sims, D. W., Squire, L., and Semmens, J. M. 2015. A new method for resolving uncertainty of energy requirements in large water breathers: the "mega-flume' seagoing swim-tunnel respirometer, *Methods in Ecology and Evolution*, 6: 668-77.

White, Craig R., Phillips, Nicole F., and Seymour, Roger S. 2006. The scaling and temperature dependence of vertebrate metabolism, *Biology Letters*, 2: 125-27.

Whitney, N. M., Lear, K. O., Gaskins, L. C., and Gleiss, A. C. 2016. The effects of temperature and swimming speed on the metabolic rate of the nurse shark (Ginglymostoma cirratum, Bonaterre), *Journal of Experimental Marine Biology and Ecology*, 477: 40-46.
